# Exploring the role of adenosine deaminase in esophageal cancer and its potential for traditional Chinese medicine intervention

**DOI:** 10.3389/fmolb.2026.1798177

**Published:** 2026-04-23

**Authors:** Xiaoling Tian, Yanhong Liu, Baochun Wang, Yubo Liu, Fengqi Liu, Chenlin Wang, Shuang Lv, Yunqi Hua, Fangrui Yin

**Affiliations:** 1 Baotou Medical College, Inner Mongolia University of Science and Technology, Baotou, Inner Mongolia, China; 2 Department of Pharmacy, Binzhou Polytechnic, Binzhou, Shandong, China; 3 Department of oncology, Baotou Cancer Hospital, Baotou, Inner Mongolia, China; 4 Department of Rheumatology, The First Affiliated Hospital of Baotou Medical College, Baotou, Inner Mongolia, China

**Keywords:** adenosine deaminase, esophageal cancer, immune microenvironment, therapeutic target, traditional Chinese medicine

## Abstract

**Introduction:**

This study aims to systematically elucidate the association of adenosine deaminase with esophageal cancer from a genetic perspective and explore its potential underlying mechanisms and intervention strategies.

**Methods:**

Through multi-level integrative analysis, two-sample Mendelian randomization analysis was performed, followed by serum validation in an independent case-control cohort. Transcriptomic analysis, co-expression network enrichment, single-cell RNA sequencing, reverse network pharmacology screening, and molecular docking simulation were conducted.

**Results:**

Mendelian randomization identified adenosine deaminase as a potential risk factor for esophageal cancer (OR = 1.23, 95% CI: 1.00–1.52). Significantly elevated serum ADA levels were validated in patients. ADA expression was upregulated in tumor tissues, and its co-expression network was significantly enriched in pathways related to “proteasome” and “protein folding.” Single-cell analysis showed high ADA expression primarily in plasma cells and plasmacytoid dendritic cells. Two potential lead compounds, catechin and flavoxanthin, were identified to stably bind to ADA.

**Discussion:**

Collectively, these findings suggest a potential role for ADA in the occurrence of esophageal cancer and highlight its possible relevance as a therapeutic target, providing new directions for early intervention and targeted therapy of esophageal cancer.

## 1 Introduction

Esophageal cancer (EC) is a malignant tumor with a high incidence and mortality rate globally (Jin et al., 2025). It poses a heavy burden on the public health system. Despite continuous advancements in comprehensive strategies such as surgery, chemoradiotherapy, and immunotherapy, the overall prognosis for patients remains unsatisfactory, with a 5-year survival rate of only about 22% (Jiang et al., 2025; He et al., 2021). Tumor recurrence and treatment resistance are the main reasons for treatment failure in EC (Heidt, 2026). Therefore, in-depth analysis of the molecular mechanisms underlying the initiation and progression of EC, and exploring new therapeutic targets and strategies based on this, is a key challenge faced by current clinical and basic research. Histologically, esophageal squamous cell carcinoma (ESCC) is the predominant subtype of EC, accounting for 90% of all cases. In this study, EC specifically refers to ESCC (Jiang et al., 2024).

In recent years, the role of the tumor metabolic microenvironment, especially the purine metabolic pathway, in tumor immune evasion and drug resistance has become a research focus (Mao et al., 2026; Liu et al., 2024). Among these pathways, the adenosine pathway serves as a core hub. In the tumor microenvironment, extracellular ATP is degraded by enzymes such as CD39 and CD73 into adenosine, which exerts a strong immunosuppressive effect; this inhibits T cell and NK cell functions, thereby creating an immune-privileged area (Allard et al., 2020; Xing et al., 2023). Adenosine deaminase (ADA), a key metabolic enzyme in this pathway, is responsible for deaminating adenosine to inosine, and it plays a complex and context-dependent role (Cristalli et al., 2001; Whitmore and Gaspar, 2016). ADA1 and ADA2, as isoenzymes, have distinct functions in cancer: ADA1 is more closely associated with tumor progression and poor prognosis (Gao et al., 2022; Kaljas et al., 2017), while ADA2 exhibits a complex dual role, potentially exerting protective effects by degrading adenosine and also promoting tumor progression through mechanisms such as enhancing M2 macrophage polarization (Ombrello et al., 2019; Ehlers and Meyts, 2025). Together, they influence tumor initiation and development by regulating adenosine signaling, immune cell interactions, and the inflammatory response (Brix et al., 2024). In addition, ADAR1, another member of the adenosine deaminase family that acts on RNA, has been reported to be highly expressed in ESCC and drives tumor progression, which is associated with poor prognosis in patients (Marônek et al., 2025). These findings suggest that the entire adenosine deaminase family may play an important but complex and underappreciated role in EC.

Although the importance of ADA in tumors is increasingly prominent, the specific mechanism of its role in the occurrence and development of EC remains unclear. Traditional observational studies find it difficult to infer the causal relationship between circulating inflammatory factors and EC due to their susceptibility to confounding factors, while Mendelian randomization (MR) uses heritable variation as an instrumental variable and provides a reliable framework for revealing the causal association between protein exposure and disease outcomes (Ference et al., 2021; Burgess et al., 2015). Building on this approach, this study employs an innovative integrated research strategy of “from cause to effect, targeting drugs” (Zhang et al., 2025). It systematically clarifies the causal role of ADA in EC through multi-omics integration analysis. This approach may also reveal new functions of ADA that are independent of classical immune metabolism. Furthermore, the study provides new perspectives for understanding the complex biological roles of this protein, empirical evidence, and candidate compounds to facilitate the development of its precision therapeutic strategies.

## 2 Methods

### 2.1 Study design

**FIGURE 1:**
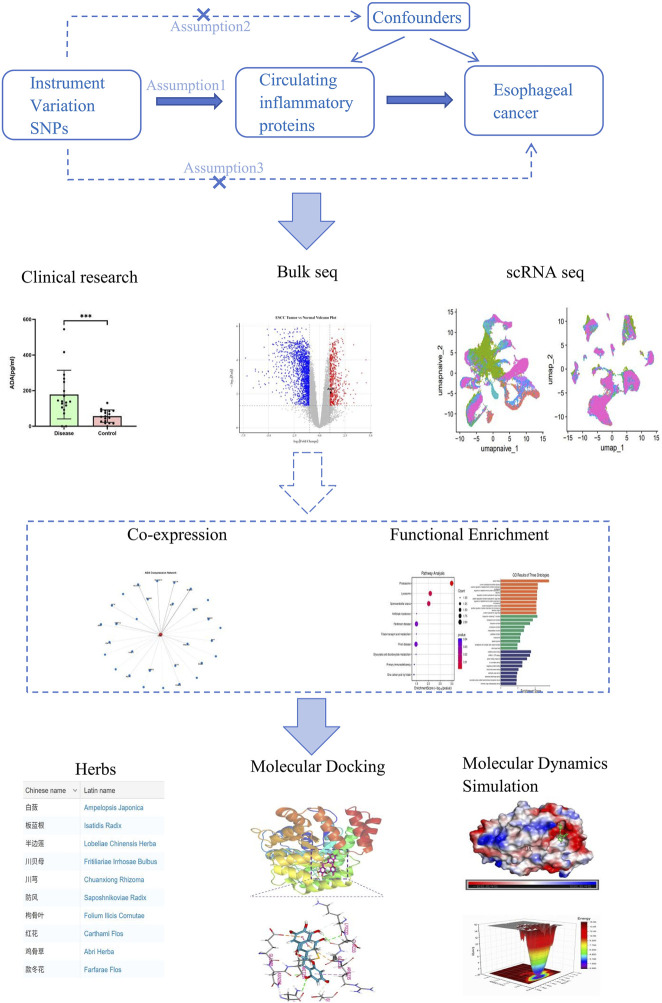
Study design overview. Schematic illustration of the multi-stage integrative analysis strategy, integrating two-sample Mendelian randomization, serological validation, bulk and single-cell transcriptomic analysis, and network pharmacology combined with molecular docking and dynamics simulations to identify adenosine deaminase as a causal risk factor and therapeutic target in esophageal cancer.

The present study adopts a multi-stage integrative analysis strategy (Figure 1). First, using publicly available genetic data, a two-sample MR analysis is conducted to assess the causal association between circulating inflammatory proteins and the risk of EC. Second, independent clinical samples are utilized for validation through serological experiments. Subsequently, bulk RNA-sequencing and single-cell transcriptomic data are integrated to elucidate the expression patterns and functional mechanisms of key molecular targets. Finally, based on the elucidated mechanisms of the targets, network pharmacology approaches combined with computational simulation methods are employed to screen and evaluate candidate compounds derived from traditional Chinese medicine.

### 2.2 Data sources

Genome-wide association study (GWAS) statistics for the circulating inflammatory proteins can be found in the GWAS catalogue under accession numbers GCST90274758 to GCST90274848 (https://www.ebi.ac.uk/gwas/studies/GCST90274758), presented in Supplementary Material S1. Single nucleotide polymorphisms (SNPs) associated with EC were obtained from the IEU Open GWAS project (https://gwas.mrcieu.ac.uk/datasets/ebi-a-GCST90018841/). This dataset included a total of 476,306 individuals, among whom 998 were esophageal cancer patients, and covered 24,194,380 SNPs.

### 2.3 Instrument Variables selection and MR analysis

MR analysis is primarily based on three core assumptions (correlation, independence, and exclusivity) (Zheng et al., 2017). Because very few independent single nucleotide polymorphisms (SNPs) reach a significance level (p < 5 × 10^−8^) in genome-wide association studies targeting this exposure factor, rendering them insufficient for effective analysis, this study adopts a relatively relaxed screening criterion (p < 1 × 10^−5^). To enhance the biological specificity of the selected instrumental variables and reduce potential pleiotropic bias, we also applied the ‘cloning distance’ rule, selecting only SNPs located within 500 kb (a total of 1 Mb) upstream and downstream of the corresponding protein-coding genes as candidate instrumental variables. Additionally, to meet the independence assumption, linkage disequilibrium filtering was performed using the European 1,000 Genomes reference panel (r^2^ < 0.1, window size 500 kb), retaining loci with a minor allele frequency greater than 0.01. The strength of the instrumental variables was assessed using the F-statistic (F > 10). Supplementary Material S2 provides the complete list of instrumental variables used for ADA analysis. Our Mendelian randomization analysis primarily estimates causal effects using the inverse variance weighting method. In addition, to assess the robustness of the results, the weighted median method and the MR-Egger method were further used for supplementary analysis. Heterogeneity was assessed using Cochran’s Q test, while horizontal pleiotropy was verified through MR-Egger intercept test and MR-PRESSO global test (Bowden et al., 2015). All MR analyses were performed using R software (version 4.3.5), with the TwoSampleMR (version 0.5.6), MendelianRandomization (version 0.9.0), and MR-PRESSO(version 1.0) packages. Visualization was performed using the ggplot2 (version 3.4.0) package.

### 2.4 Independent validation of serum protein levels

To validate the results of genetic analysis at the protein level, the study included an independent group of case-control samples. The study included 18 diagnosed EC patients and 18 age- and sex-matched healthy controls from August 2024 to May 2025. The study protocol was approved by the ethics committee of Baotou Cancer Hospital, and all participants signed written informed consent. Fasting peripheral blood was collected from participants, and serum was separated for analysis. The concentration of ADA in the serum was quantified using a specific human ADA enzyme-linked immunosorbent assay (ELISA) kit (Wuhan Boster Biological Technology Co., Ltd, Product Number: EK1446). All procedures strictly followed the manufacturer’s instructions. Samples were diluted at a ratio of 1 part sample to 2 parts diluent, as determined by preliminary experiments. Each sample was set in three separate wells, with a standard curve included on each plate for quality control. The final concentration was calculated using the absorbance values measured at a wavelength of 450 nm and the standard curve. Continuous variables were expressed as median (interquartile range). Since the data did not conform to a normal distribution, comparisons between groups were performed using the non-parametric Mann-Whitney U test. A p-value <0.05 was considered statistically significant. All statistical analyses and charting were completed using GraphPad Prism 9.5.0 software.

### 2.5 Transcriptome data analysis

#### 2.5.1 Bulk RNA data analysis

Download the dataset GSE213565 from the Gene Expression Omnibus (GEO) database, and filter the dataset to include EC tissues and their paired adjacent normal tissue samples. Use the limma R package for differential expression analysis, applying a significance threshold of P < 0.05 after false discovery rate (FDR) correction, and |log_2_ (fold change)| ≥ 1 to select differentially expressed genes.

#### 2.5.2 Co-expression and functional enrichment analysis

Based on the above bulk RNA data, the Pearson correlation coefficient of expression between all genes and ADA was calculated. Subsequently, a gene set significantly co-expressed with ADA was identified (|r| > 0.6, and FDR <0.05). The clusterProfiler R package was used to perform Gene Ontology (GO) functional term and Kyoto Encyclopedia of Genes and Genomes (KEGG) pathway enrichment analysis on this gene set, with a significance threshold set at FDR <0.05.

#### 2.5.3 Single-cell RNA sequencing data analysis

We obtained the single-cell RNA sequencing dataset GSE188900 from the GEO database and performed data processing using the Seurat R package (v4.0). Quality control filtered out low-quality cells based on the following criteria: the number of genes detected per cell was less than 200 or greater than 5,000, or the mitochondrial gene proportion exceeded 10%. After normalization using the LogNormalize method, we identified the top 2,500 highly variable genes for principal component analysis. To correct for batch effects across different patient samples, we applied Harmony using the first 30 principal components, with patient sample origin as the batch variable. Cell clustering was performed using a graph-based algorithm on the corrected embedding, and dimensionality reduction was visualized using Uniform Manifold Approximation and Projection (UMAP) based on the same corrected dimensions. Finally, cell type annotation was conducted using the SingleR R package (Chen et al., 2025).

### 2.6 Screening and evaluation of candidate compounds from traditional Chinese medicine

#### 2.6.1 Targeted screening

Using a target-oriented strategy, all traditional Chinese medicines related to the target protein “Adenosine Deaminase” were retrieved from the Traditional Chinese Medicine Systems Pharmacology Database (TCMSP). The number of active ingredients in each traditional Chinese medicine that simultaneously meets the criteria of oral bioavailability (OB) ≥ 30% and drug-likeness (DL) ≥ 0.18 was calculated for preliminary ranking. Subsequently, a comprehensive evaluation was conducted by combining traditional Chinese medicine treatment principles—clearing heat and detoxifying; promoting blood circulation; and removing blood stasis—with relevant anti-tumor pharmacological literature evidence to determine the core candidate traditional Chinese medicines. Finally, based on structural diversity, adequacy of existing pharmacological evidence, and drug-like property parameters, up to three active ingredients were selected from each traditional Chinese medicine as candidate compounds for subsequent analysis (Yang et al., 2018).

#### 2.6.2 Molecular docking

The crystal structure of human ADA was obtained from the RCSB Protein Data Bank (PDB ID:3IAR, with coordinates: X = 6.692, Y = −3.339, Z = 0.089 Å),and the 3D structures of candidate compounds were retrieved from the PubChem database. AutoDockTools was used to prepare the protein and ligand files by removing water molecules, adding polar hydrogens, and assigning charges. Molecular docking was performed using AutoDock Vina, with the docking grid centered on the known catalytic active site of ADA and the grid size set to 50 Å × 50 Å × 50 Å. The conformation exhibiting the lowest binding free energy was selected as the optimal binding mode for further analysis (Du et al., 2020). To validate the reliability of the docking protocol, pentostatin, a well-characterized FDA-approved ADA inhibitor, was included as a positive control using the same docking parameters (Wang and Quiocho, 1998).

#### 2.6.3 Molecular dynamics simulation

To evaluate the binding stability of the docking complex, molecular dynamics simulations were performed on the protein-ligand complex exhibiting the most favorable binding free energy (Du et al., 2020; Ma et al., 2020). The simulations were conducted using GROMACS version 2022.2 software. The protein and ligand were assigned the AMBER14SB and GAFF2 force fields, respectively. The system was placed in a cubic box under periodic boundary conditions, solvated with the TIP3P water model, and physiological ionic concentrations of Na^+^ and Cl^−^ ions were added to neutralize the system’s charge.

The simulation process included energy minimization, NVT, and NPT equilibration. The final production simulation was conducted for 100 ns at 298 K and 1 bar. Trajectory analysis included the calculation of root mean square deviation (RMSD), root mean square fluctuation (RMSF), radius of gyration (Rg), and hydrogen bond occupancy analysis to characterize structural stability and interactions. In addition to trajectory analysis, the binding free energy was estimated using the MM-PBSA method. Principal component analysis (PCA) and free energy landscape (FEL) analyses were used to describe the main motion patterns and conformational stability of the complex. All analyses and visualizations were completed using GROMACS built-in tools, VMD, and PyMOL.

## 3 Results

### 3.1 ADA was identified as a causal risk inflammatory protein for EC

**FIGURE 2:**
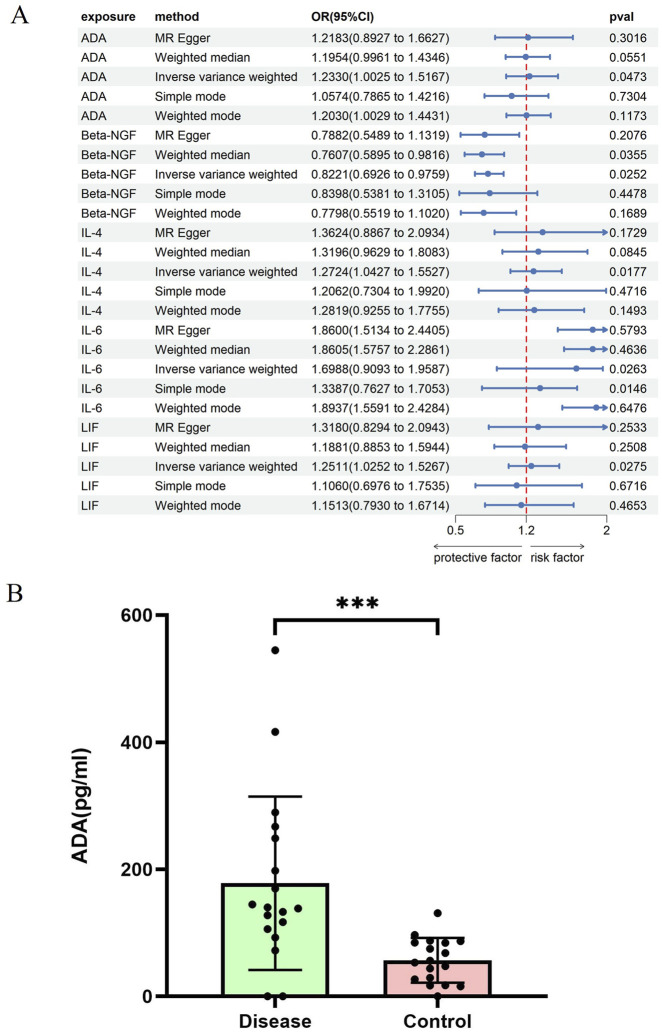
Causal association between circulating inflammatory proteins and esophageal cancer risk. **(A)** Forest plot of Mendelian randomization results for circulating inflammatory proteins with potential causal associations with esophageal cancer. **(B)** Comparison of serum ADA levels between esophageal cancer patients (n = 18) and healthy controls (n = 18) measured by ELISA.

Through two-sample MR analysis of 91 circulating inflammatory proteins, 5 proteins with potential causal associations with EC were initially identified (Figure 2A). Among them, Beta-NGF (OR = 0.82, 95% CI: 0.69–0.98, P = 0.025) and IL-6 (OR = 0.70, 95% CI: 0.51–0.96, P = 0.026) are protective factors. ADA (OR = 1.23, 95% CI: 1.00–1.52, P = 0.047), IL-4 (OR = 1.23, 95% CI: 1.00–1.52, P = 0.047), and LIF (OR = 1.25, 95% CI: 1.03–1.53, P = 0.027) are risk factors. Furthermore, the sensitivity analysis did not detect significant heterogeneity or pleiotropy, supporting the robustness of the results. The results can be found in Supplementary Material S3.

To validate the above genetic findings at the protein level, we conducted an independent case-control serological study using ELISA. Among the five candidate proteins, only the median concentration of ADA in the serum of EC patients (139.1 U/L) was significantly higher than that of healthy controls (54.7 U/L,Mann-Whitney U test, P = 0.0001) (Figure 2B); this difference was consistent with the risk direction inferred by MR. The serum levels of the other proteins were either inconsistent with the direction of genetic inference or showed no significant differences between groups. This evidence supports the role of ADA as a potential causal risk factor for EC and a promising serum biomarker from both genetic association and experimental measurement perspectives.

### 3.2 ADA is highly expressed in EC tissue and is related to the protein homeostasis regulatory network

**FIGURE 3:**
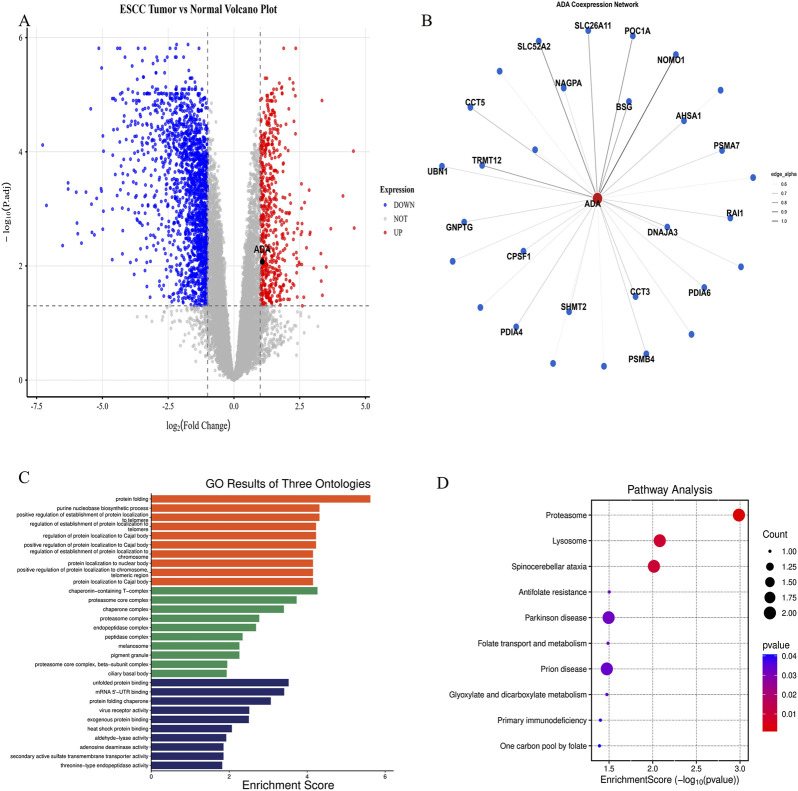
ADA expression and functional enrichment analysis in esophageal cancer tissues. **(A)** Volcano plot of differentially expressed genes between esophageal cancer tissues and paired adjacent normal tissues from the GSE213565 dataset. **(B)** Network visualization of the top 20 core genes co-expressed with ADA. **(C)** GO functional enrichment analysis of ADA co-expressed genes, showing biological process (BP), cellular component (CC), and molecular function (MF) categories. **(D)** KEGG pathway enrichment analysis of ADA co-expressed genes.

Using the GEO dataset GSE213565, we found that the mRNA expression level of ADA in EC tissue is higher than that in paired, adjacent normal tissue (log_2_FC = 1.08). This increase is consistent with the elevated ADA levels observed in serum (Figure 3A).

To further explore the potential functional context of ADA in EC, we conducted a co-expression analysis based on the same dataset. A total of 5,320 genes significantly positively correlated with ADA expression were identified (Pearson’s r > 0.6, FDR <0.01), among which the top 20 core genes (such as AHSA1, BSG, CCT3) constituted a central hub of this functional network (Figure 3B). KEGG pathway enrichment analysis showed that these co-expressed genes were most significantly enriched in the proteasome pathway (corrected P-value = 0.027) and showed enrichment trends in pathways such as lysosomes, folate metabolism, and primary immunodeficiency (corrected P-value <0.1) (Figure 3D). GO functional enrichment analysis further confirmed that these genes mainly function in the biological processes (BP) of protein folding (corrected P-value = 0.0012) and purine biosynthetic process (corrected P-value = 0.0036). At the cellular component (CC) level, they are located in the T-complex containing chaperone proteins and proteasome core complex. At the molecular function (MF) level, they are involved in unfolded protein binding and chaperone-mediated protein folding activity (Figure 3C). Collectively, these results indicate that ADA is closely associated with the functional network involved in protein folding and degradation machinery, suggesting a potential role in protein homeostasis in EC tumor cells.

### 3.3 Single-cell analysis reveals high expression of ADA in plasma cells and plasmacytoid dendritic cells

**FIGURE 4:**
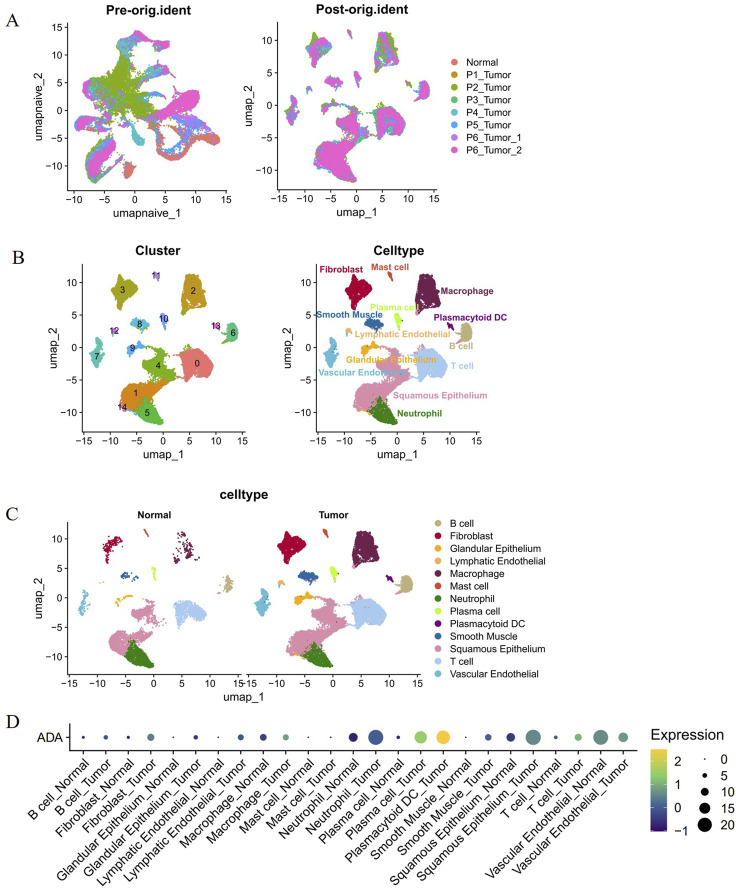
Single-cell transcriptomic landscape of normal and esophageal cancer tissues. **(A)** UMAP visualization colored by sample origin, showing uniform distribution of cells across patients and normal tissue after batch effect correction. **(B)** UMAP visualization colored by annotated cell types, identifying 13 major cell populations. **(C)** UMAP visualization split by tissue type (normal vs. tumor), illustrating compositional differences in the tumor microenvironment. **(D)** Dot plot showing ADA expression across cell types, confirming highest expression in plasma cells and pDCs.

To explore the cellular sources of the aforementioned function-associated gene signals, we further analyzed the expression pattern of ADA at single-cell resolution. After batch effect correction (Figure 4A), we identified 13 major subpopulations through cell clustering—including B cells, plasma cells, plasmacytoid dendritic cells (pDC), and squamous epithelial cells, among others (Figure 4B). ADA expression was assessed across these cell types, revealing expression in various cell populations, with particularly prominent expression in plasma cells and pDC (Figures 4C,D). This precise cellular expression localization indicates that ADA’s role in the EC tumor microenvironment may be primarily mediated by specific immune cell subpopulations. This finding connects tissue-level functional pathways to the specific immune cell subpopulations responsible for executing these functions.

### 3.4 Screening and validation of traditional Chinese medicine lead compounds targeting ADA

Based on the established ADA target from the aforementioned research, we employed a reverse network pharmacology strategy to screen potential inhibitors from TCMSP (Table 1). Considering the number of high-quality active ingredients, the efficacy of traditional Chinese medicines, and evidence from anti-tumor literature, we selected Ampelopsis japonica, Lobelia chinensis Herba, and Carthamus flos for in-depth analysis, and screened out eight active ingredients that meet pharmacokinetic criteria (Table 2).

**TABLE 1:** Ranking of high-quality active components in traditional Chinese medicines targeting ADA (top 10).

Traditional Chinese medicine	Chinese name	Number of high-quality active ingredients (OB ≥ 30% and DL ≥ 0.18)	Number of active ingredients selected
Ampelopsis Japonica	白蔹	39	3
Isatidis Radix	板蓝根	22	-
Lobeliae Chinensis Herba	半边莲	22	3
Fritiliariae Irrhosae Bulbus	川贝母	18	-
Chuanxiong Rhizoma	川芎	18	-
Saposhnikoviae Radix	防风	17	-
Folium Ilicis Cornutae	枸骨叶	15	-
Carthami Flos	红花	13	2
Abri Herba	鸡骨草	13	-
Farfarae Flos	款冬花	13	-

**TABLE 2:** Molecular docking binding energy of active ingredients with ADA.

Receptor	Ligand ID	Ligand	Traditional Chinese medicine	Afffnity (kcal/mol)
ADA	MOL000569	Digallate	Ampelopsis Japonica	−7.228
	MOL000492	(+)-Catechin	Ampelopsis Japonica	−8.157
	MOL000098	Quercetin	Ampelopsis Japonica	−7.666
	MOL009009	(+)-Medioresinol	Lobeliae Chinensis Herba	−7.037
	MOL002341	Hesperetin	Lobeliae Chinensis Herba	−7.527
	MOL012216	Norlobelanine	Lobeliae Chinensis Herba	−7.177
	MOL002712	6-Hydroxykaempferol	Carthami Flos	−7.797
	MOL002680	Flavoxanthin	Carthami Flos	−8.367

**FIGURE 5:**
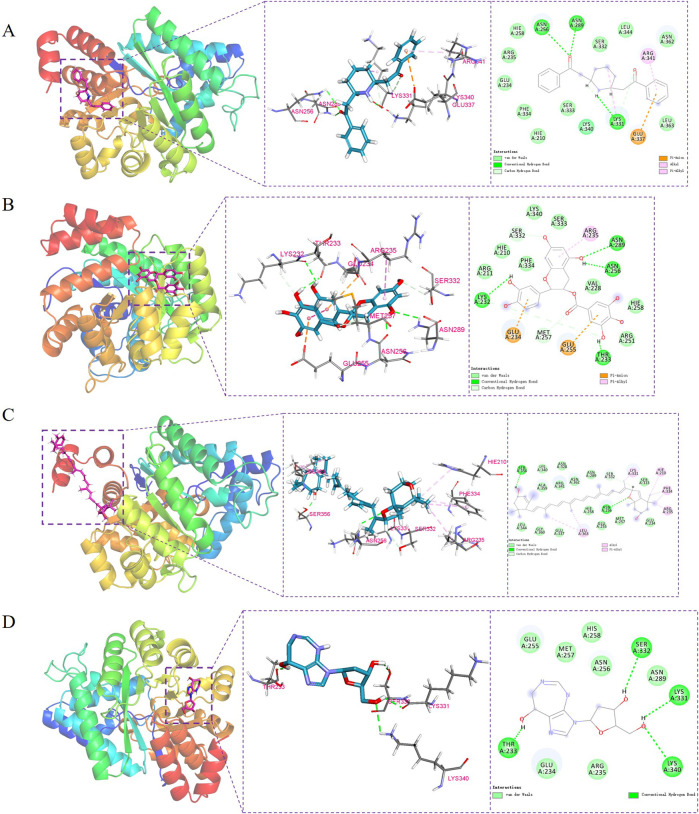
Molecular docking of candidate compounds and a positive control with ADA. **(A)** Norlobelanine; **(B)** Catechin; **(C)** Flavoxanthin; **(D)** Pentostatin.

**FIGURE 6:**
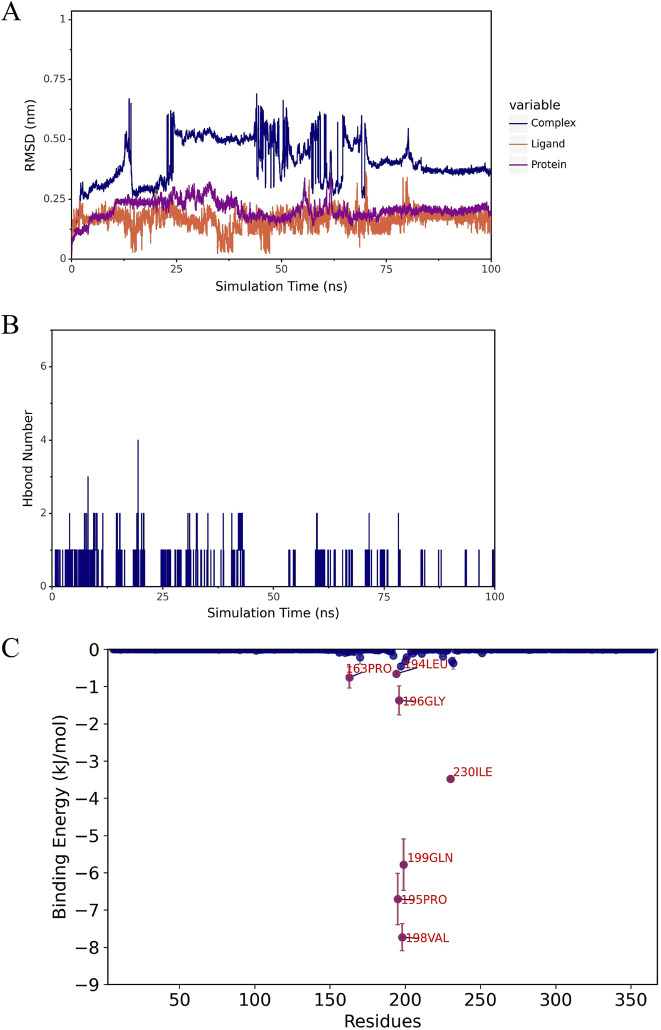
Molecular dynamics simulation of ADA in complex with candidate compounds. Norlobelanine from Lobelia chinensis Herba; **(A)** RMSD of the complex, ADA protein, and ligand; **(B)** number of hydrogen bonds; **(C)** amino acid energy contribution (MM-PBSA).

**FIGURE 7:**
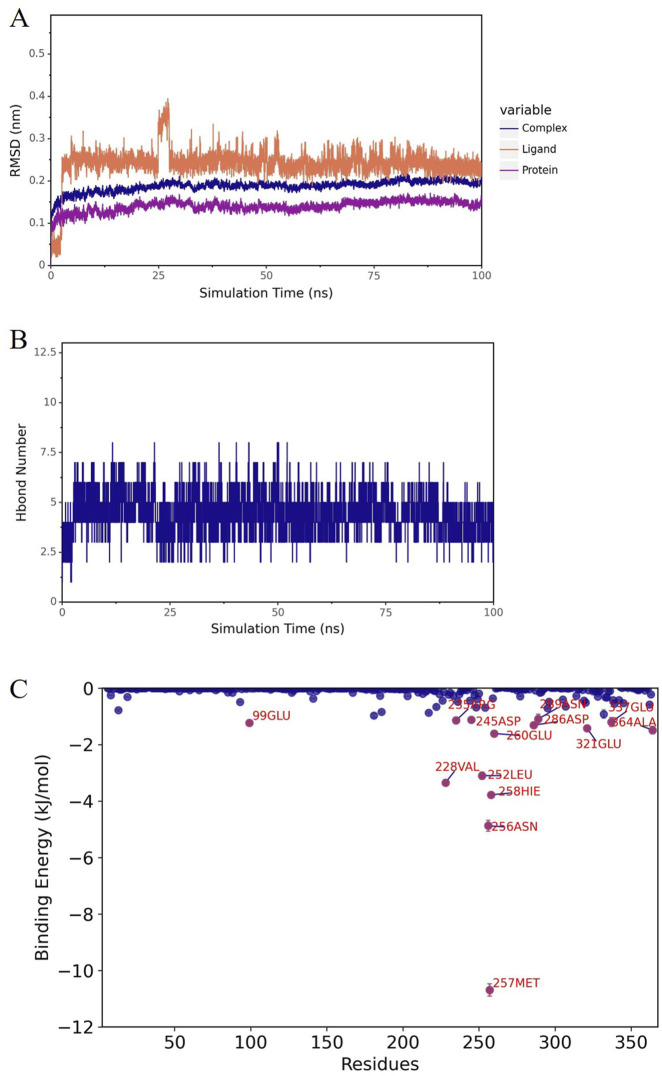
Molecular dynamics simulation of ADA in complex with candidate compounds. Catechin from Ampelopsis japonica; **(A)** RMSD of the complex, ADA protein, and ligand; **(B)** number of hydrogen bonds; **(C)** amino acid energy contribution (MM-PBSA).

**FIGURE 8:**
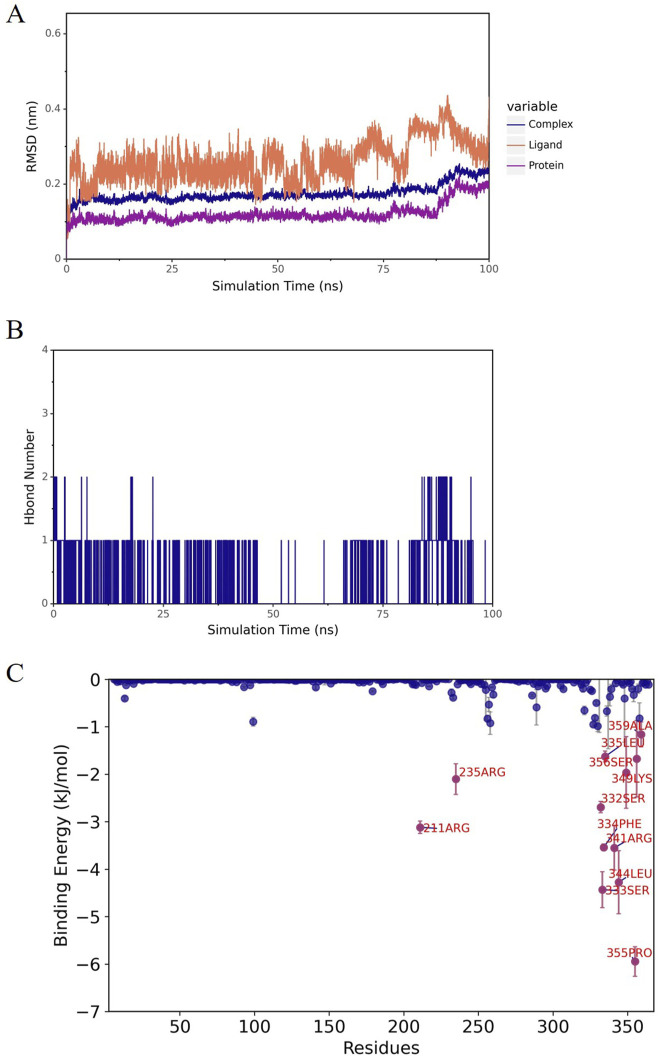
Molecular dynamics simulation of ADA in complex with candidate compounds. Flavoxanthin from Carthami flos; **(A)** RMSD of the complex, ADA protein, and ligand; **(B)** number of hydrogen bonds; **(C)** amino acid energy contribution (MM-PBSA).

Molecular docking analysis identified three compounds with optimal binding free energy: norlobelanine, isolated from Lobeliae Chinensis Herba; catechin, derived from Ampelopsis Japonica; and flavoxanthin, obtained from Carthami Flos (Figure 5). A subsequent 100 ns molecular dynamics simulation evaluated their binding stability. The root mean square deviation of the norlobelanine-ADA complex fluctuated dramatically, with the ligand dissociating early from the binding pocket, indicating unstable binding (Figure 6). In contrast, the catechin-ADA and flavoxanthin-ADA complexes remained stable throughout the simulation. Catechin primarily binds by forming a stable hydrogen bond network with residues such as ASP-229 and GLU-234 (Figure 7). Flavoxanthin, on the other hand, relies mainly on strong hydrophobic interactions with residues such as LEU-363 and PHE-334, with its MM-PBSA-calculated binding free energy (−91.23 ± 0.22 kJ/mol) being the lowest (most favorable) among the three compounds (Figure 8). Therefore, catechin and flavoxanthin were identified as promising lead compounds derived from traditional Chinese medicine that can stably target ADA, providing a novel chemical scaffold for subsequent anti-esophageal cancer drug development.

Molecular docking results showed that pentostatin binds spontaneously to the hydrophobic pocket of ADA. The ligand is anchored primarily through conventional hydrogen bonds with residues THR233, SER332, LYS331, and LYS340, along with extensive van der Waals interactions with residues including GLU234, GLU255, MET257, ASN256, ASN229, ARG235, and HIS258 (Figure 5). This binding pattern is consistent with the known inhibitory mechanism of pentostatin (Wang and Quiocho, 1998). The successful docking and stable interaction mode of this positive control confirm that our molecular docking protocol can reliably capture the binding characteristics between ADA and its inhibitors, thereby providing methodological support for the reliability of this study.

## 4 Discussion

This study systematically reveals the complex role of ADA in EC beyond traditional understanding, by integrating MR causal inference, multi-omics analysis, and reverse network pharmacology. We established ADA as a potential causal risk factor for EC from a genetic perspective and characterized its non-classical pro-cancer associations at tissue, cellular, and molecular network levels; subsequently, we completed the screening of lead compounds targeting this pathway. We actively constructed a comprehensive evidence chain from “risk discovery” to “intervention target,” which offers a new perspective for understanding the immunometabolic etiology of EC and facilitates the development of precise therapeutic strategies. Notably, the genetic association analysis in this study relies primarily on data derived from European populations, whereas the incidence rate of esophageal cancer (especially ESCC) is substantially higher in Asian regions. Consequently, caution is warranted when extrapolating conclusions regarding the utility of ADA as a biomarker or therapeutic target to Asian populations, and future validation in independent cohorts from high-incidence areas in Asia is essential.

MR and serological analysis established that ADA is a potential risk factor for EC, which contradicts the traditional understanding that ADA exerts antitumor effects by degrading the immunosuppressive molecule adenosine (O'Dwyer et al., 1988). This difference may be because this study measured total ADA, failing to distinguish between ADA1 and ADA2. Given that ADA1 and ADA2 often play opposing roles in carcinoma (Gao et al., 2022): high expression of ADA1 is usually positively correlated with poor prognosis in various cancers (Song and Song, 2025). In contrast, ADA2 is often associated with better prognosis or unique immune regulatory functions (Wang L. et al., 2021). The positive correlation we observed between total ADA and EC risk likely reflects the dominant tumor-promoting role of ADA1. Thus, in the EC population, carcinogenic signaling pathways may selectively upregulate ADA1, or the activity of ADA1 may account for a larger proportion of total ADA, thereby driving the overall increased risk.

Secondly, the functional enrichment analysis in this study provides a new potential mechanistic explanation for the tumor-promoting role of ADA that extends beyond classical immune metabolism. The core of its network is not enriched in the adenosine metabolism pathway but is significantly enriched in the “proteasome” and “protein folding” pathways. This suggests that ADA (especially ADA1) may be involved in endometrial cancer through its association with the proteostasis network of tumor cells. Proteostasis is an essential process that enables cells to cope with various internal and external stresses, including carcinogenic signals and chemotherapy-induced selective pressure, and its dysregulation is a hallmark of malignancies (Hommen et al., 2022). Therefore, ADA might indirectly influence proteasome activity through its metabolites by affecting cellular energy and nutrient-sensing pathways (such as AMPK/mTOR), potentially contributing to the clearance of proteins damaged by chemotherapy drugs and thereby supporting cancer cell survival and resistance (Wang Z. et al., 2021; Lee et al., 2010; Zhan et al., 2023). However, it is crucial to note that the aforementioned mechanisms are currently primarily based on hypotheses derived from bioinformatics enrichment analysis, and direct experimental evidence is still lacking. The potential role of ADA in regulating protein homeostasis to affect esophageal carcinoma progression, as well as the underlying molecular mechanisms, still requires rigorous validation via genetic manipulations (e.g., ADA knockdown or overexpression) in cell and animal models, coupled with protein degradation dynamics analysis.

We also found that the role of ADA in EC may not be limited to extracellular metabolism but may extend to an association with intracellular proteostasis processes and might serve as a hub linking immune metabolism and intracellular protein quality control mechanisms. Existing studies have shown that the rapid proliferation of tumor cells and the activation of immune cells both rely on efficient protein synthesis and quality control mechanisms (Luo and Lee, 2013). The proteasome is a key complex for antigen processing, and its function directly affects MHC-I class antigen presentation (Rana et al., 2023). Therefore, ADA’s association with these pathways may reflect its potential involvement in maintaining proteostasis in tumor cells through direct or indirect means, potentially helping them resist ER stress and thus enhancing survival (Gao et al., 2021). ADA might also affect both the processing and presentation efficiency of tumor antigens, thereby interfering with CD8^+^T-cell recognition (Darragh and Karam, 2022). Finally, it could have a regulatory effect on the proteostasis of infiltrating immune cells, such as activated T cells and pDCs, affecting their functional integrity (Gao et al., 2021). Based on these findings, we propose the hypothesis that in the EC microenvironment, ADA may function as a potential dual node of “immune metabolism–proteostasis.” On one hand, it may contribute to an immunosuppressive metabolic environment by degrading adenosine; on the other hand, it might support a pro-tumor microenvironment through its association with proteostasis. This hypothesis provides a new conceptual framework for understanding the complex mechanisms of ADA; however, its validity still needs to be validated through future functional experiments.

Single-cell sequencing analysis precisely locates the high expression of ADA in plasma cells and pDCs, a significant breakthrough in understanding the cellular basis of its potential pro-tumor function. This finding suggests that ADA may be involved in tumor immune regulation by modulating the function of these specific immune cells. However, it is important to emphasize that the current analysis is limited to characterizing expression patterns at the transcriptional level, which is a correlative finding. The specific functions of ADA in these cells—such as whether it modulates the type I interferon secretion capacity of pDCs or disrupts antibody synthesis and protein homeostasis in plasma cells—still require direct validation through specific genetic manipulations and functional experiments targeting primary immune cells. Both cell types play complex and often controversial roles in the tumor microenvironment (TME) (Ou et al., 2025). pDCs are the main producers of type I interferon (IFN-I), but they often exhibit functional tolerance in tumors, and can even promote immunosuppression by secreting regulatory cytokines (Asselin-Paturel and Trinchieri, 2005; Zhou et al., 2021). Our data show that the specific enrichment of ADA in pDCs may be closely related to their impaired function. Similarly, the prominent expression of ADA in plasma cells is also highly enlightening. Plasma cells are the terminal effector cells responsible for antibody secretion. Their survival and function heavily depend on an efficient endoplasmic reticulum and robust protein synthesis/folding capabilities (Göritzer et al., 2025; Gaudette and Allman, 2021). The overexpression of ADA here might disrupt the metabolic and secretory balance of plasma cells by interfering with local purine metabolism or by potentially affecting endoplasmic reticulum-associated degradation (ERAD) (Bhagavatham et al., 2021). This could lead to abnormal secretion of non-specific antibodies and the production of proinflammatory effectors, or indirectly influence the inflammatory tone of the TME through the “endoplasmic reticulum stress-unfolded protein response” axis (Calsolaro and Edison, 2016). Therefore, we speculate that ADA may contribute to the formation of a tumor-promoting microenvironment by influencing the functional programs of these specific immune cells at both systemic (elevated serum ADA) and local (TME cell-specific expression) levels.

Based on the understanding of the above associations, we targeted ADA and its associated pathways for rational virtual screening from traditional Chinese medicine resources. The identified candidate compounds, catechin and flavoxanthin, both showed stable binding with the ADA protein in molecular docking and dynamics simulations, although the binding modes differed. Catechin primarily binds to the catalytic pocket through a hydrogen bond network, which may inhibit the canonical enzymatic activity of ADA (Baranwal et al., 2022). Flavoxanthin, on the other hand, mainly relies on hydrophobic interactions to bind to the potential protein interaction interface, which may be more conducive to interfering with the non-canonical functions of ADA or the formation of protein complexes (Safe et al., 2021). These two compounds, which exhibit different mechanisms of action, provide useful chemical probes for future experimental models to validate ADA’s “enzyme” function and “homeostatic regulation” hypotheses, respectively. More importantly, their complementary mechanisms—targeting distinct functional sites—provide a preliminary chemical starting point for developing combination therapy strategies, such as simultaneously relieving immune suppression and disrupting tumor cell tolerance, or for designing bifunctional molecules. Current research remains limited to computer simulation. Confirming whether these compounds effectively target ADA and exert anti-tumor effects requires determining the half-maximal inhibitory concentration (IC50) of these candidates via *in vitro* enzyme inhibition assays, followed by validation of their efficacy and targeting stability in cellular and animal models (Jeyaraj et al., 2025).

## 5 Limitations of the study

Although this study proposes a coherent and insightful scientific hypothesis, several limitations need to be addressed in future work. First, regarding the generalizability of the findings, this study is primarily based on data from European populations, which may not fully reflect the genetic characteristics of Asian populations in high-incidence areas of esophageal cancer (especially the ESCC subtype). Future research must validate the predictive value of ADA as a genetic risk factor and serum biomarker in large, independent Asian cohorts to ensure its clinical relevance across different ethnicities. Second, concerning the proposed non-classical mechanisms, although multi-omics analysis suggests a close association between ADA and proteasome activity and protein folding pathways, direct *in vivo* and *in vitro* experimental evidence is currently lacking to establish a causal regulatory role. Future studies should employ gene knockdown or overexpression experiments in esophageal cancer cell lines to determine whether ADA directly contributes to the regulation of cellular protein homeostasis. Third, regarding the cell specificity of ADA function, although single-cell analysis revealed high expression of ADA in plasma cells and pDCs, the exact functional consequences in these specific immune cell subsets remain to be explored. Future research needs to directly demonstrate how ADA expression in these cells leads to immune dysregulation through gene manipulation and functional experiments targeting primary immune cells. Additionally, existing techniques are unable to differentiate between the activities of serum ADA1 and ADA2, necessitating the development of specific detection methods to dissect the individual contributions of each subtype to esophageal cancer risk and progression. Finally, regarding the screening of lead compounds, the results of virtual screening require rigorous pharmacological validation, including *in vitro* enzyme inhibition assays to determine IC50 values, as well as cellular and *in vivo* animal model studies to confirm their efficacy, target engagement and stability, and therapeutic potential against esophageal cancer, before proceeding to the preclinical development stage.

## 6 Conclusion

In summary, this study established ADA as a potential genetic causal risk factor for esophageal cancer through multidimensional integrative analysis and proposed a novel hypothesis that ADA may be involved in tumor progression through its association with protein homeostasis. Single-cell level analysis focused the enrichment of ADA expression in plasma cells and pDCs, providing refined cellular atlas clues for its potential role in the tumor microenvironment. Screening of lead compounds derived from traditional Chinese medicine targeting this molecule provided a new foundation for chemical optimization for subsequent drug development. The main contribution of this study lies in constructing a complete evidence chain from “risk discovery” to “intervention target” through multi-omics integrative analysis and proposing a series of scientific hypotheses that can be validated by subsequent experiments. Future research should: 1) validate the predictive value of ADA in independent cohorts from high-incidence areas in Asia; 2) investigate the molecular mechanism underlying the association between ADA and protein homeostasis through *in vitro* and *in vivo* functional experiments; 3) elucidate the cell-specific functions of ADA in pDCs and plasma cells; 4) validate the efficacy and targeting of lead compounds through enzyme activity inhibition experiments and animal models. This work not only expands the understanding of the complex role of ADA in tumor biology but also provides new theoretical insights and practical foundations for mechanistic research, biomarker discovery, and targeted therapy strategy design in EC.

## Data Availability

The original contributions presented in the study are included in the article/Supplementary Material, further inquiries can be directed to the corresponding authors.
