# Supplementary material for: Exploring the role of adenosine deaminase in esophageal cancer and its potential for traditional Chinese medicine intervention
Source: Front Mol Biosci. 2026 Apr 23;13:1798177. doi: 10.3389/fmolb.2026.1798177 (PMC13149097; doi:10.3389/fmolb.2026.1798177)
Supplement: Supplementary file 1 [file DataSheet3.zip › Data Sheet 6.PDF]

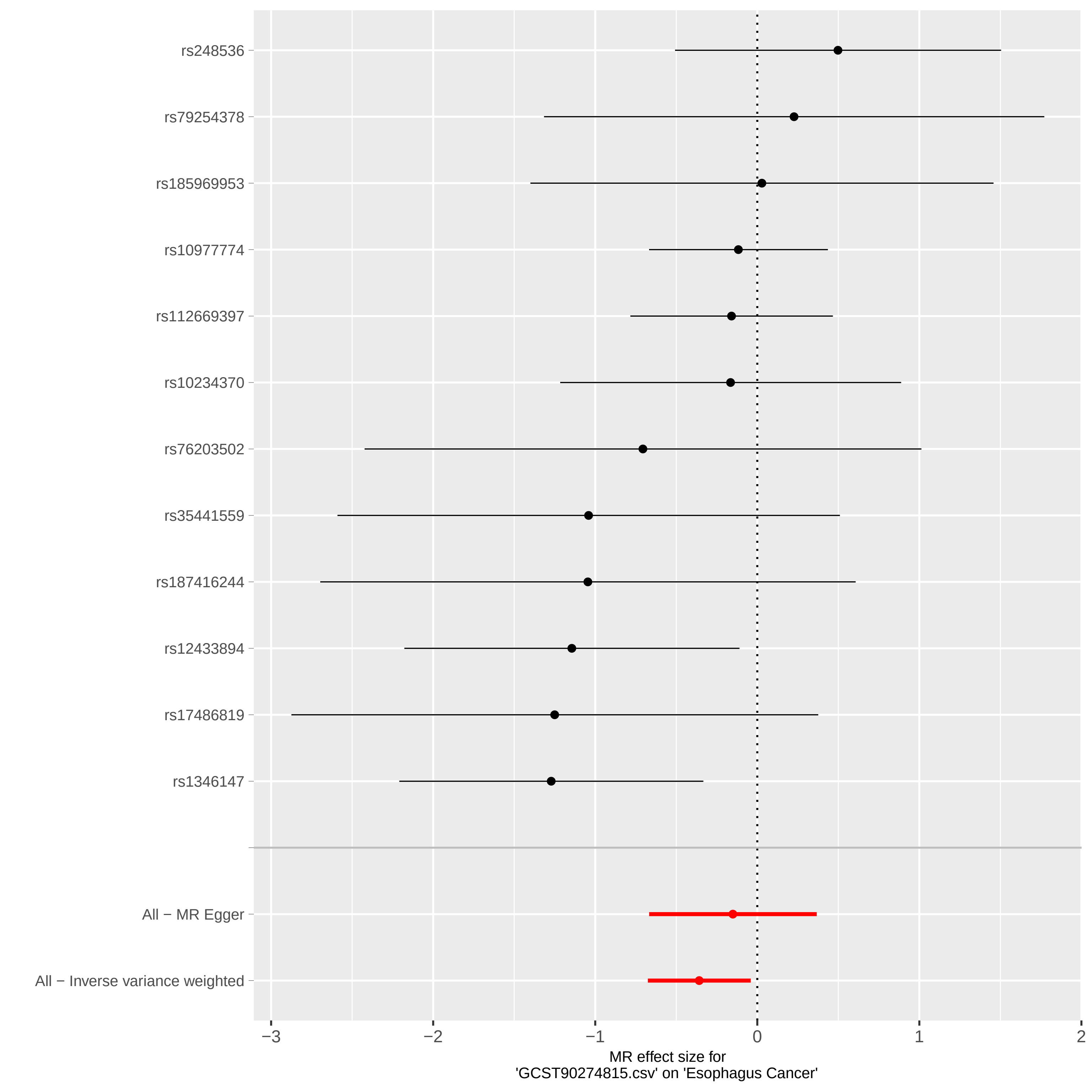
rs248536
rs79254378
rs185969953
rs10977774
rs112669397
rs10234370
rs76203502
rs35441559
rs187416244
rs12433894
rs17486819
rs1346147
All − MR Egger
All − Inverse variance weighted
−3
−2
−1
0
1
2
MR effect size for
'GCST90274815.csv' on 'Esophagus Cancer'

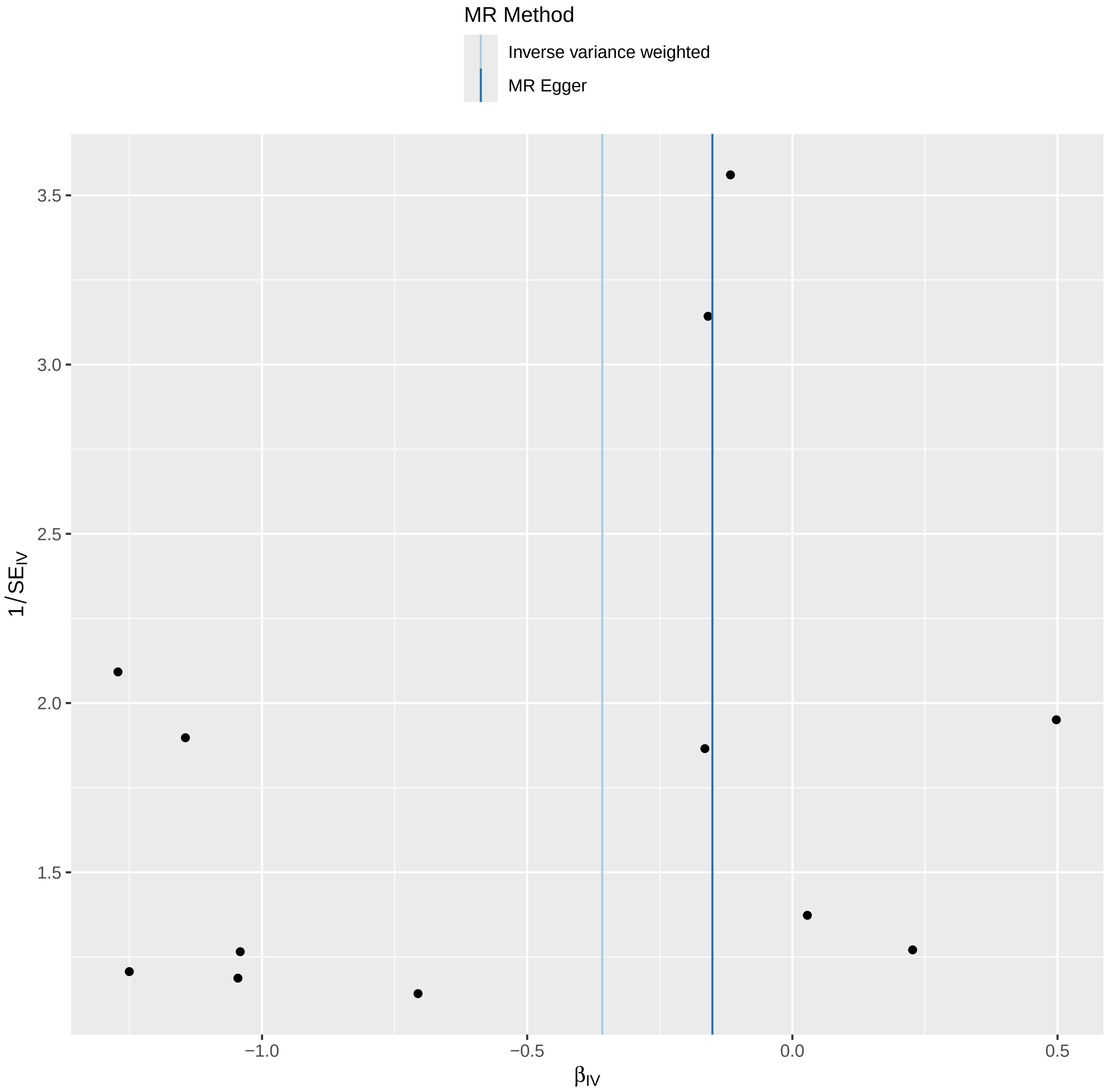

MR Method
Inverse variance weighted
MR Egger
3.5
3.0
2.5
2.0
1.5
$1/SE_{IV}$
−1.0
−0.5
0.0
0.5
β_{IV}

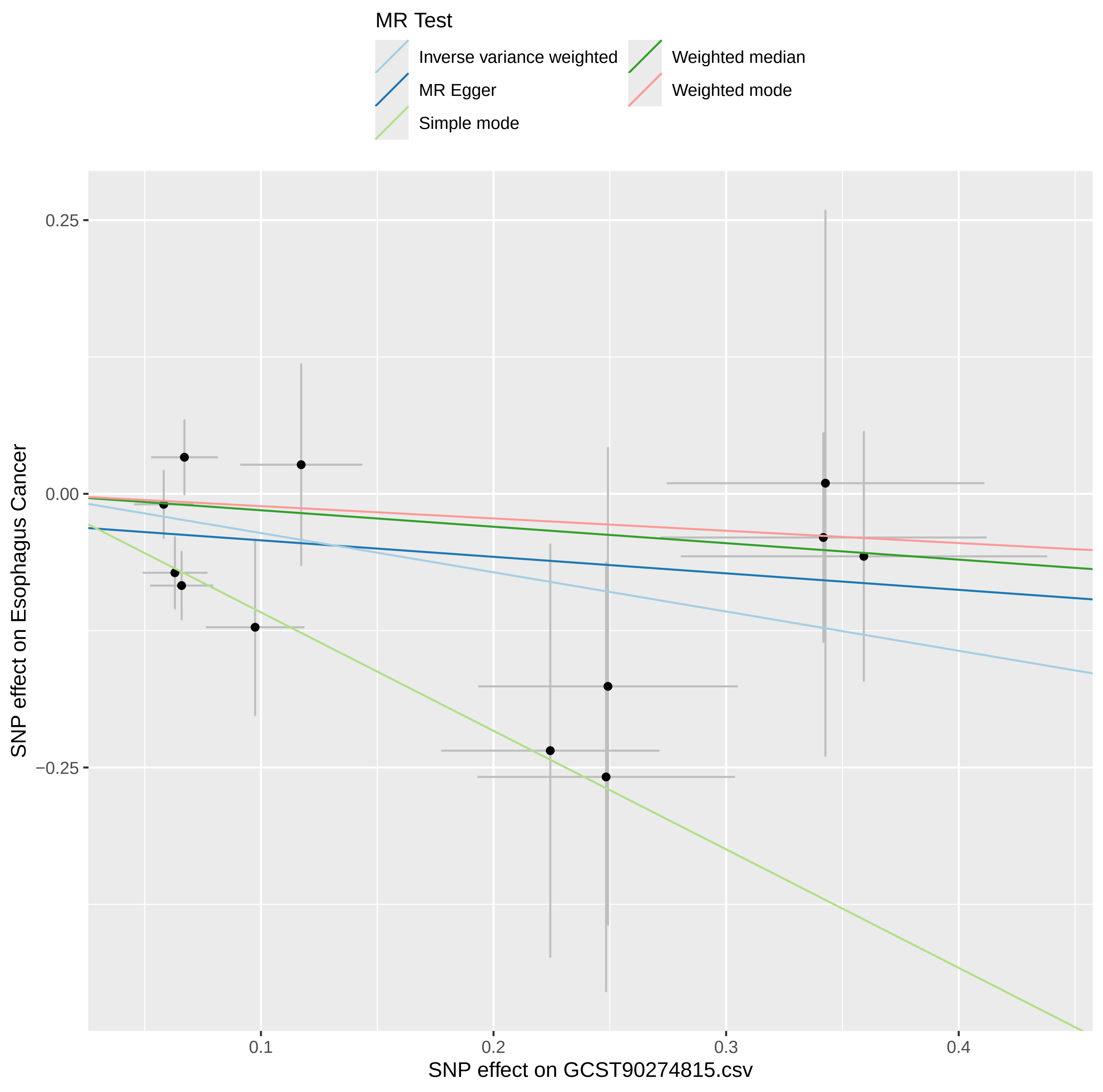

MR Test
Inverse variance weighted
MR Egger
Simple mode
Weighted median
Weighted mode
0.25
0.00
−0.25
0.1
0.2
0.3
0.4
SNP effect on Esophagus Cancer
SNP effect on GCST90274815.csv

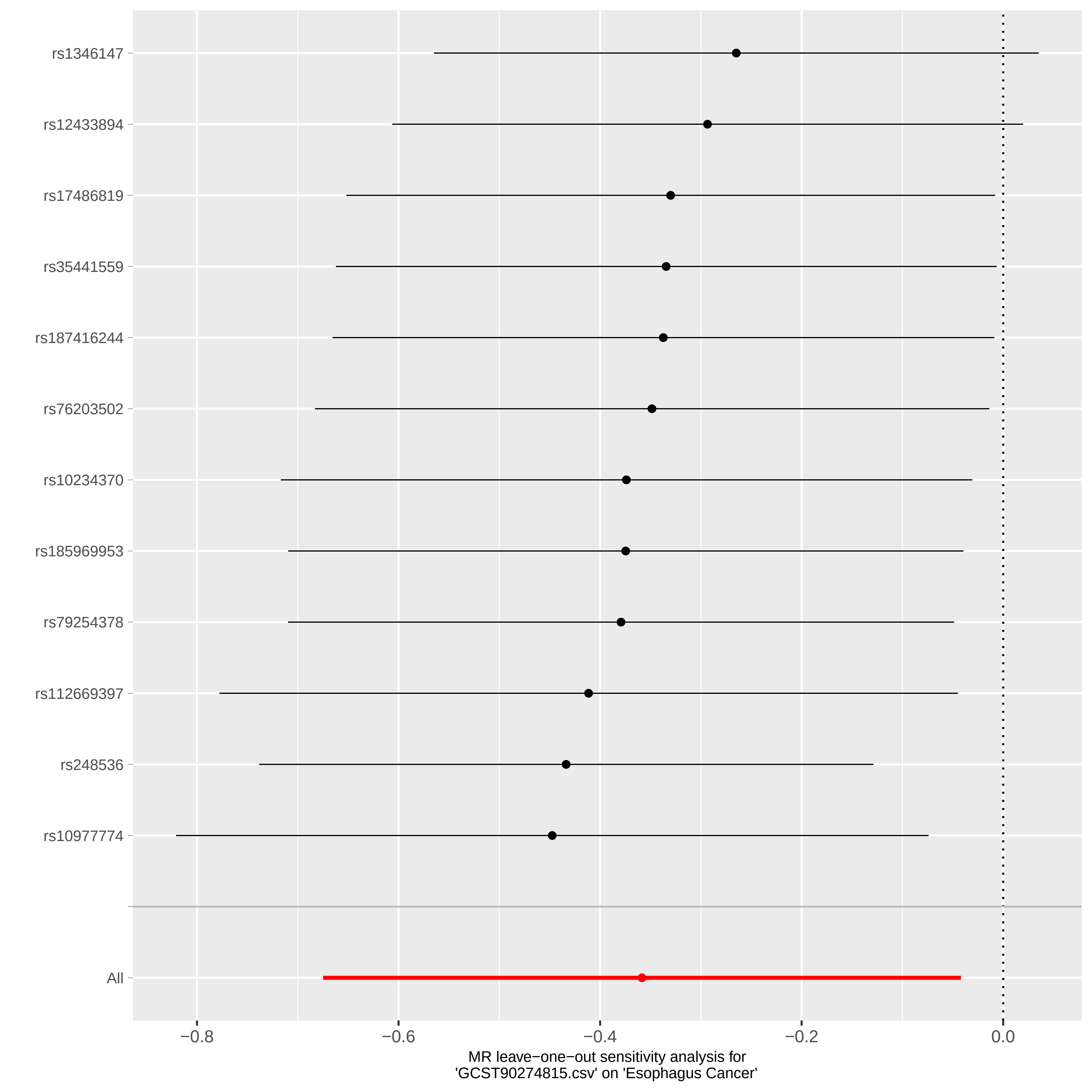

rs1346147
rs12433894
rs17486819
rs35441559
rs187416244
rs76203502
rs10234370
rs185969953
rs79254378
rs112669397
rs248536
rs10977774
All
−0.8
−0.6
−0.4
−0.2
0.0
MR leave−one−out sensitivity analysis for
'GCST90274815.csv' on 'Esophagus Cancer'
