# Supplementary material for: Exploring the role of adenosine deaminase in esophageal cancer and its potential for traditional Chinese medicine intervention
Source: Front Mol Biosci. 2026 Apr 23;13:1798177. doi: 10.3389/fmolb.2026.1798177 (PMC13149097; doi:10.3389/fmolb.2026.1798177)
Supplement: Supplementary file 1 [file DataSheet3.zip › Data Sheet 5.PDF]

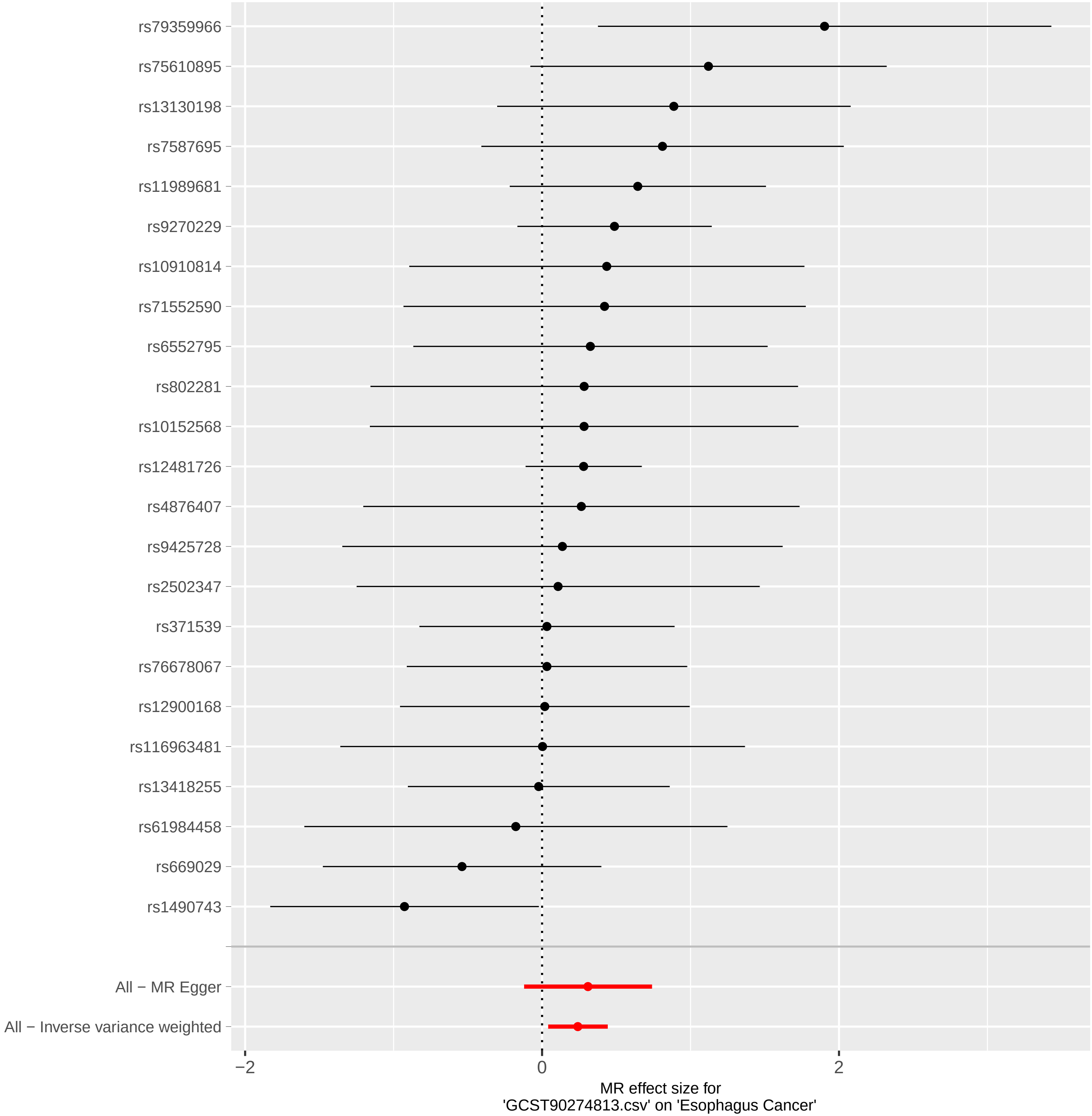

rs79359966
rs75610895
rs13130198
rs7587695
rs11989681
rs9270229
rs10910814
rs71552590
rs6552795
rs802281
rs10152568
rs12481726
rs4876407
rs9425728
rs2502347
rs371539
rs76678067
rs12900168
rs116963481
rs13418255
rs61984458
rs669029
rs1490743
All − MR Egger
All − Inverse variance weighted
−2
0
2
MR effect size for
'GCST90274813.csv' on 'Esophagus Cancer'

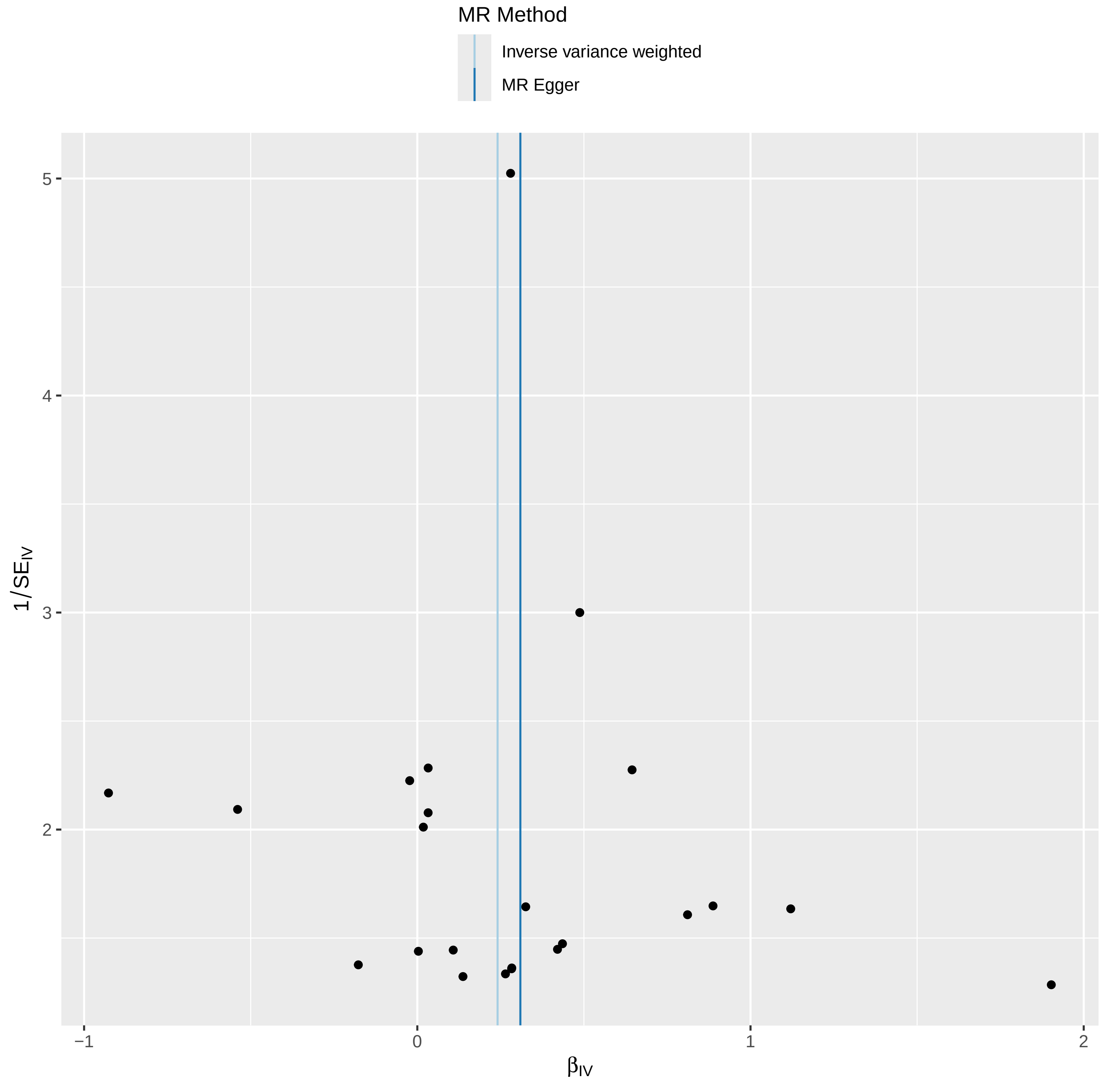

MR Method
Inverse variance weighted
MR Egger
1/SE_IV
5
4
3
2
−1
0
1
2
β_IV

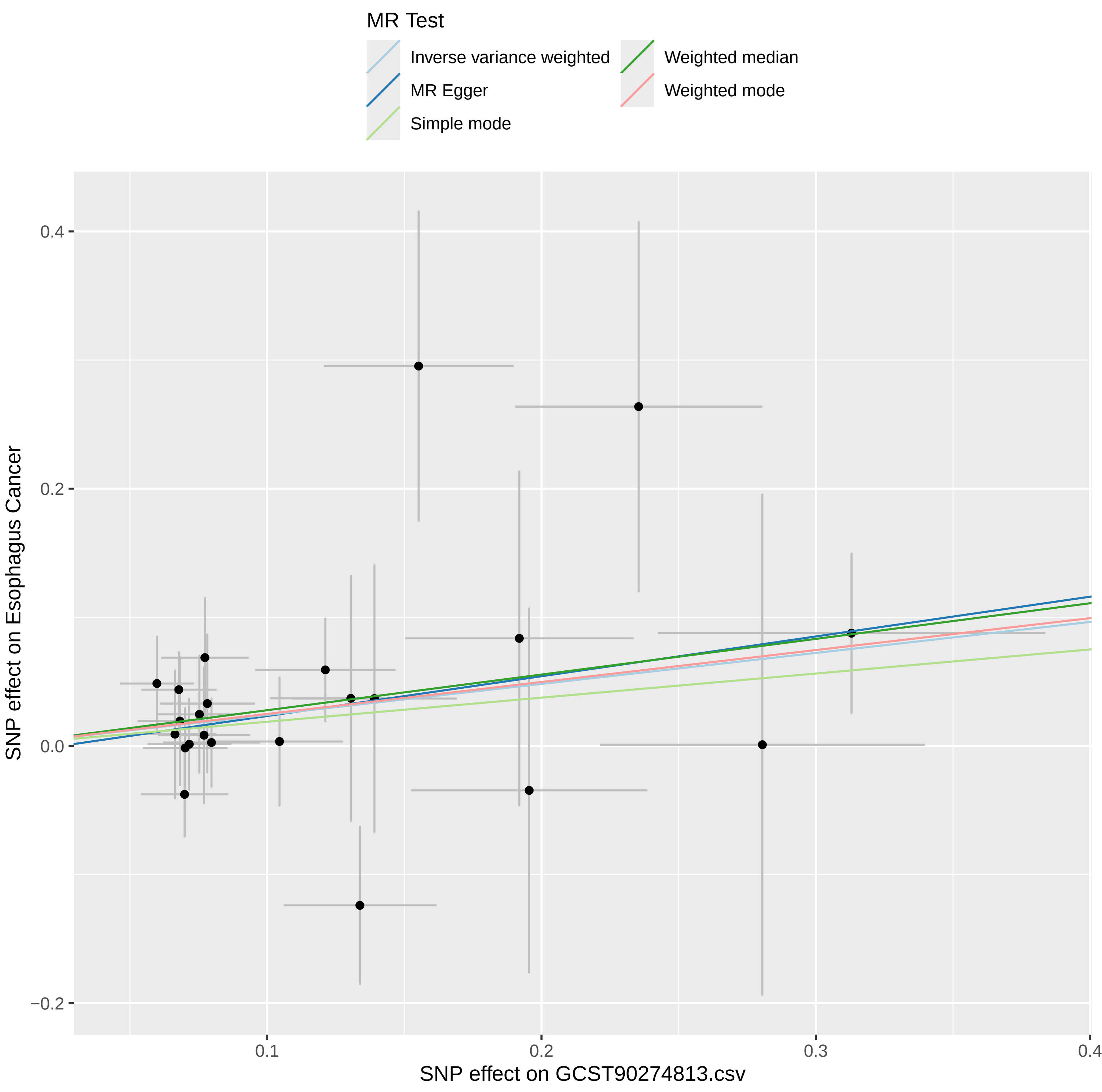

MR Test
Inverse variance weighted
MR Egger
Simple mode
Weighted median
Weighted mode
SNP effect on Esophagus Cancer
0.4
0.2
0.0
−0.2
0.1
0.2
0.3
0.4
SNP effect on GCST90274813.csv

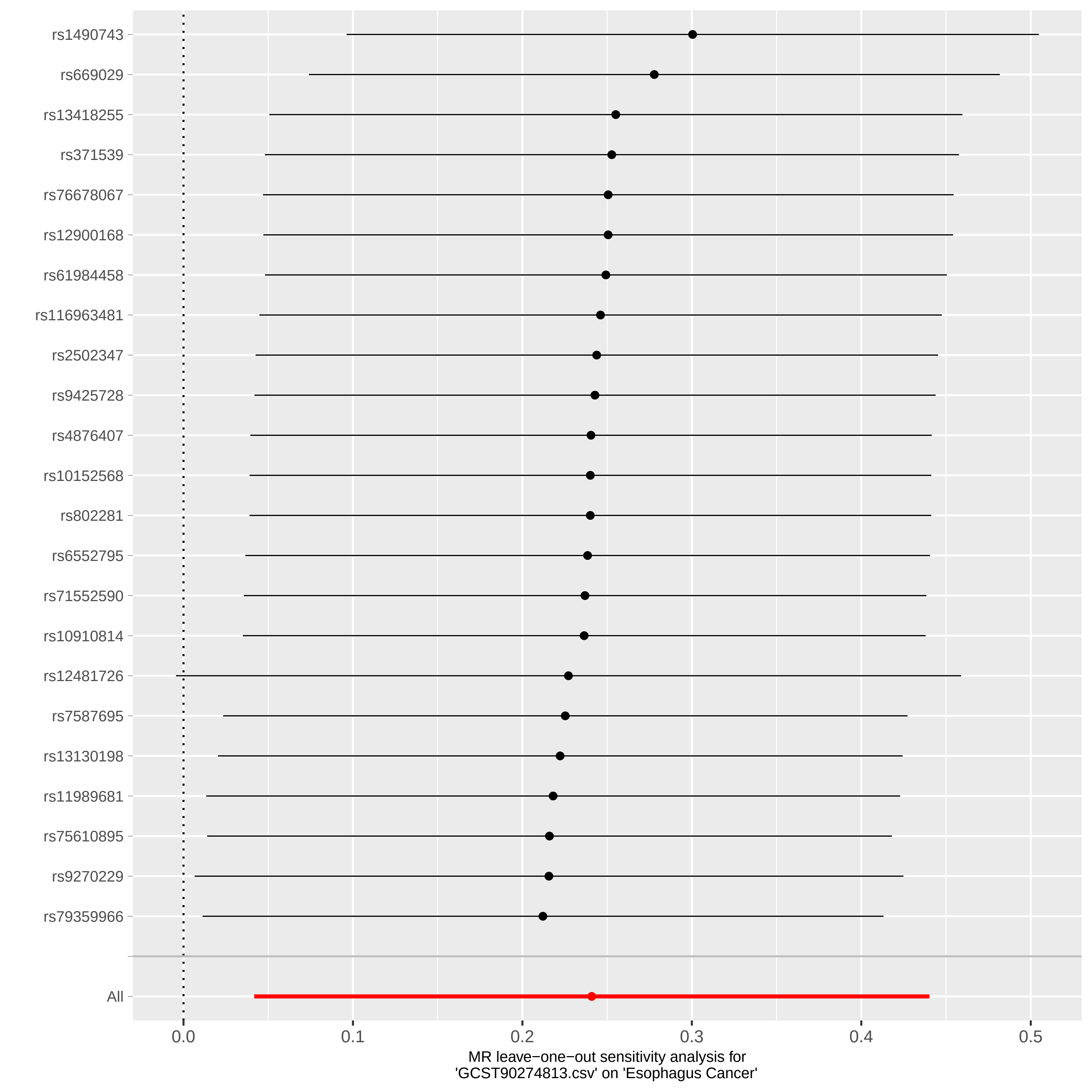
rs1490743
rs669029
rs13418255
rs371539
rs76678067
rs12900168
rs61984458
rs116963481
rs2502347
rs9425728
rs4876407
rs10152568
rs802281
rs6552795
rs71552590
rs10910814
rs12481726
rs7587695
rs13130198
rs11989681
rs75610895
rs9270229
rs79359966
All
0.0
0.1
0.2
0.3
0.4
0.5
MR leave−one−out sensitivity analysis for
'GCST90274813.csv' on 'Esophagus Cancer'
