# Supplementary material for: Exploring the role of adenosine deaminase in esophageal cancer and its potential for traditional Chinese medicine intervention
Source: Front Mol Biosci. 2026 Apr 23;13:1798177. doi: 10.3389/fmolb.2026.1798177 (PMC13149097; doi:10.3389/fmolb.2026.1798177)
Supplement: Supplementary file 1 [file DataSheet3.zip › Data Sheet 4.PDF]

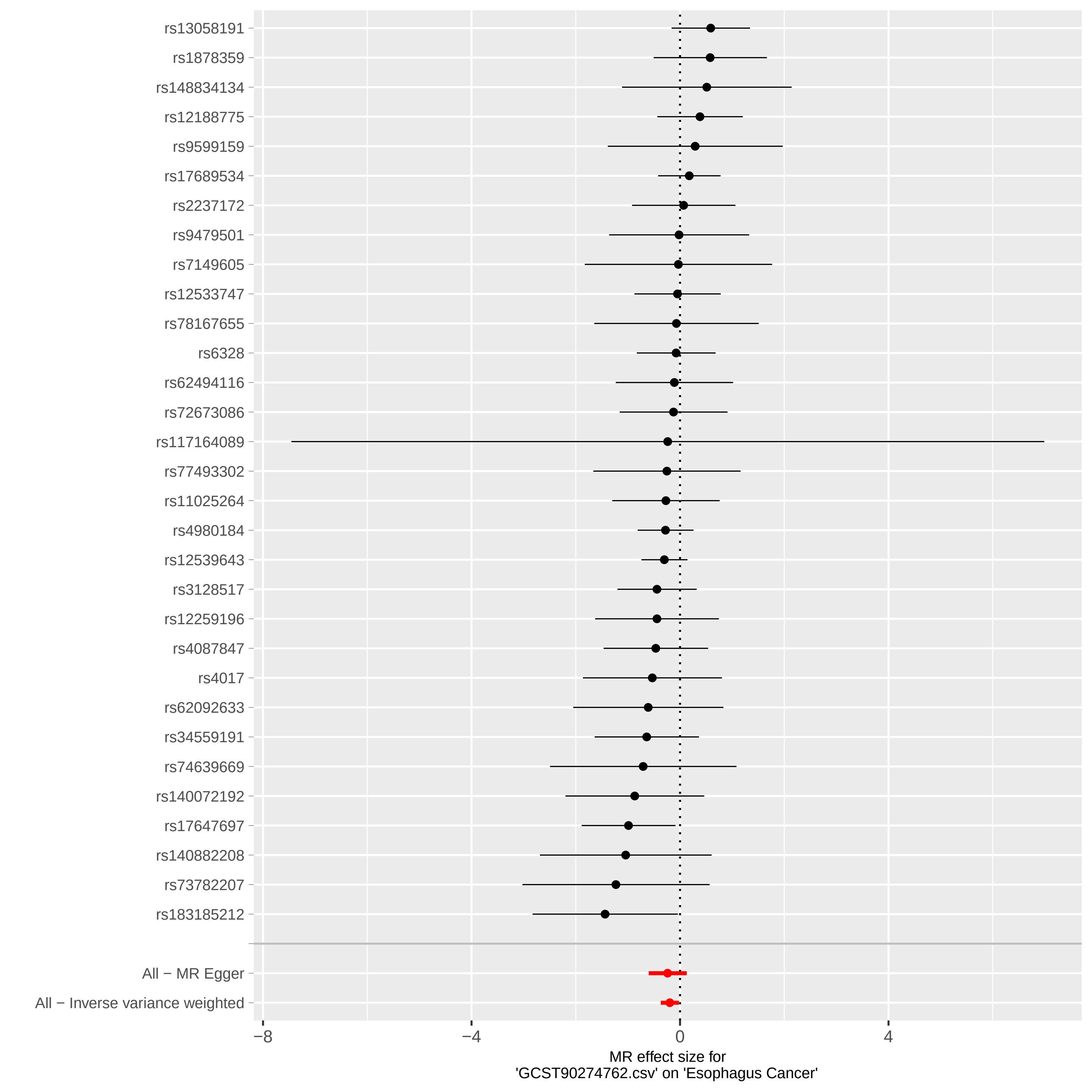
rs13058191
rs1878359
rs148834134
rs12188775
rs9599159
rs17689534
rs2237172
rs9479501
rs7149605
rs12533747
rs78167655
rs6328
rs62494116
rs72673086
rs117164089
rs77493302
rs11025264
rs4980184
rs12539643
rs3128517
rs12259196
rs4087847
rs4017
rs62092633
rs34559191
rs74639669
rs140072192
rs17647697
rs140882208
rs73782207
rs183185212
All − MR Egger
All − Inverse variance weighted
−8
−4
0
4
MR effect size for
'GCST90274762.csv' on 'Esophagus Cancer'

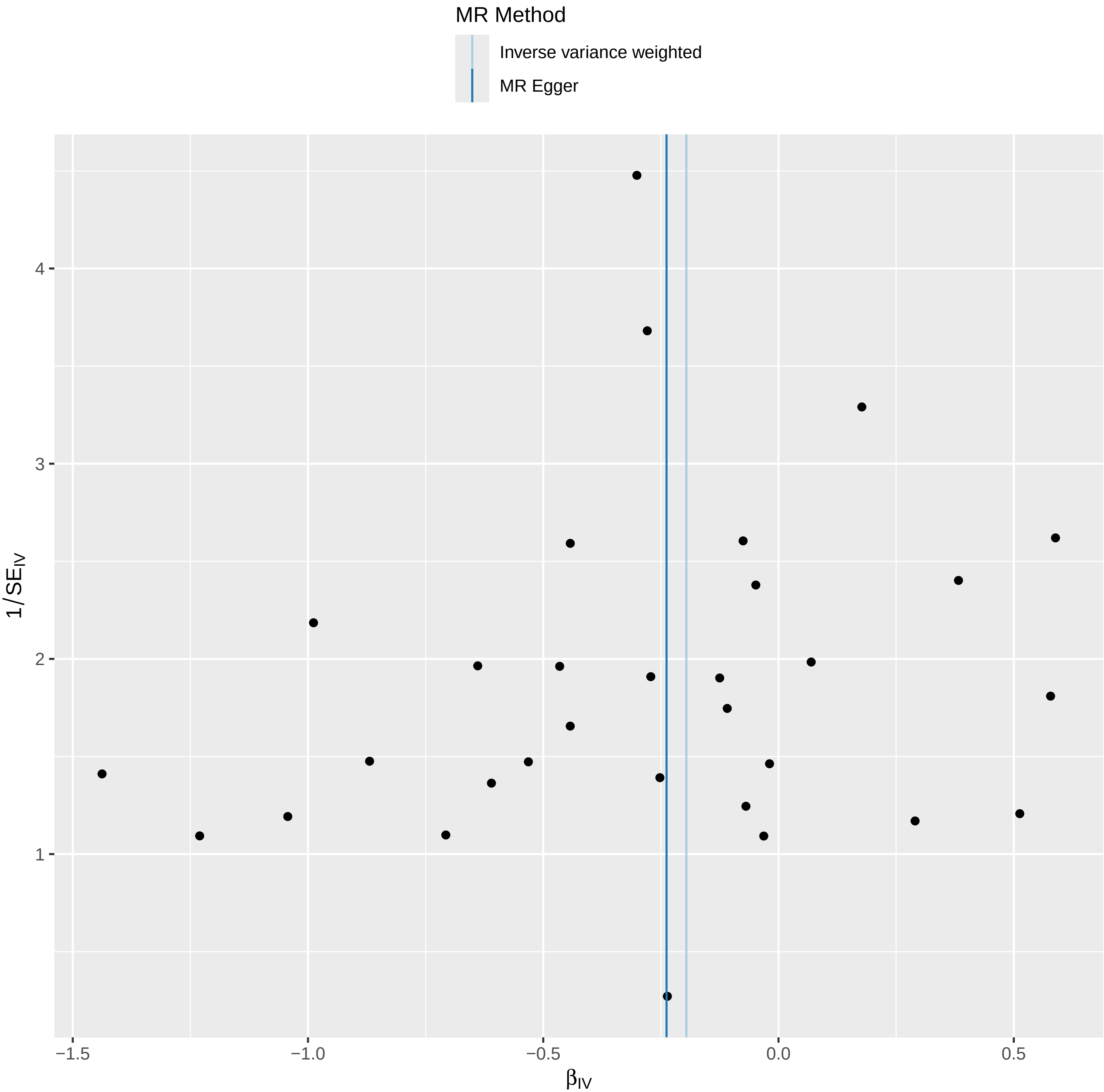

MR Method
Inverse variance weighted
MR Egger
4
3
2
1
1/SE_IV
−1.5
−1.0
−0.5
0.0
0.5
β_IV

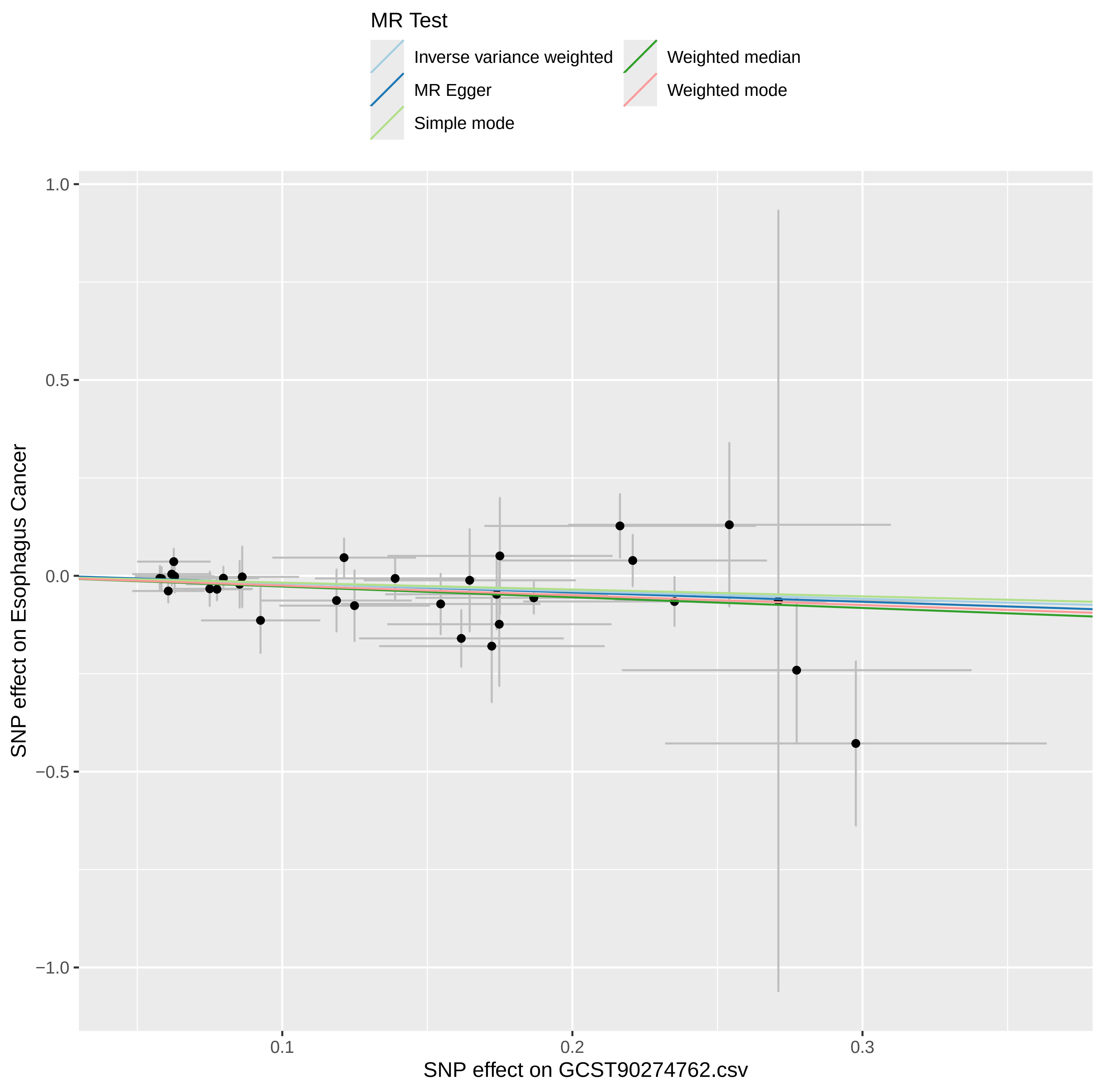
MR Test
Inverse variance weighted
MR Egger
Simple mode
Weighted median
Weighted mode
1.0
0.5
0.0
−0.5
−1.0
0.1
0.2
0.3
SNP effect on Esophagus Cancer
SNP effect on GCST90274762.csv

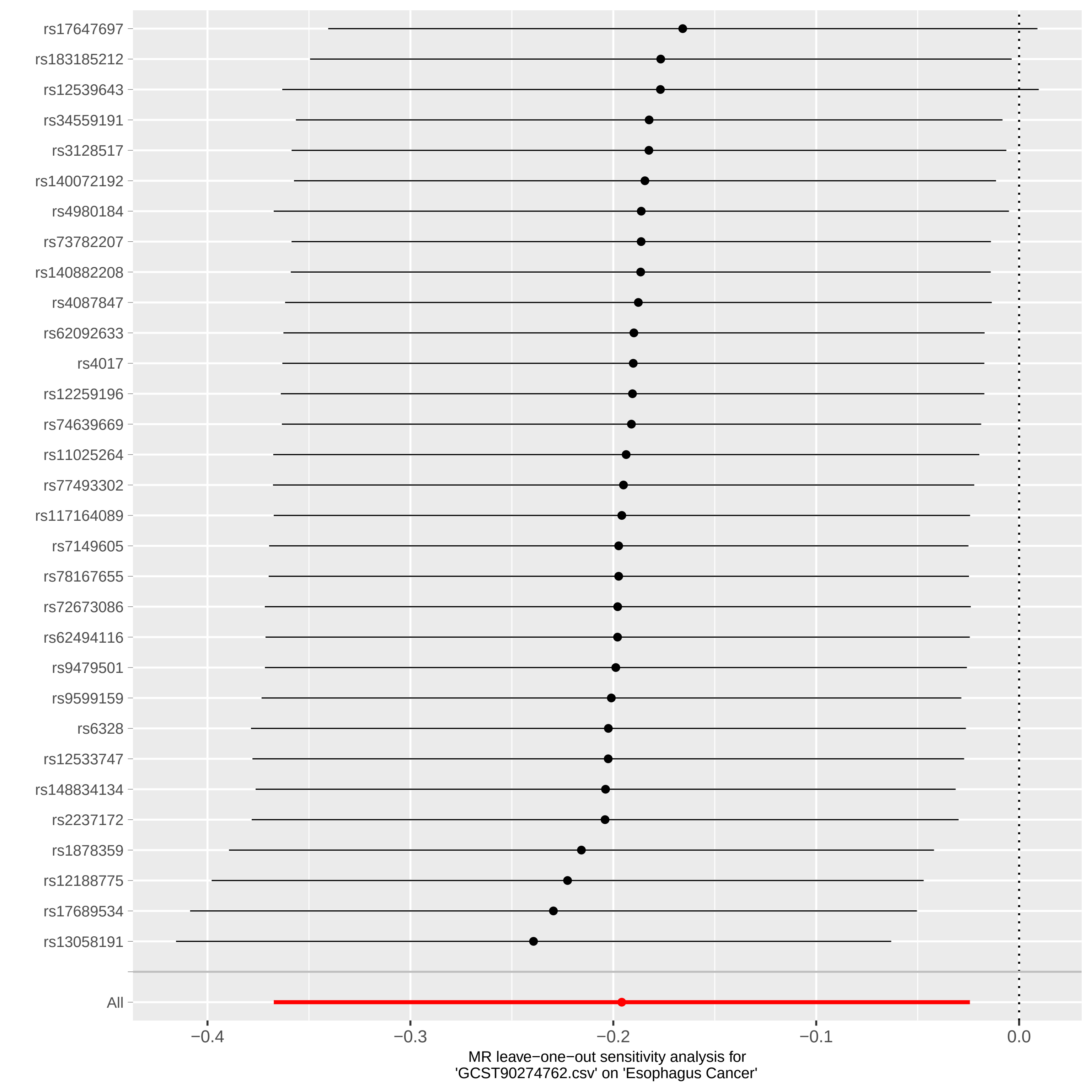
rs17647697
rs183185212
rs12539643
rs34559191
rs3128517
rs140072192
rs4980184
rs73782207
rs140882208
rs4087847
rs62092633
rs4017
rs12259196
rs74639669
rs11025264
rs77493302
rs117164089
rs7149605
rs78167655
rs72673086
rs62494116
rs9479501
rs9599159
rs6328
rs12533747
rs148834134
rs2237172
rs1878359
rs12188775
rs17689534
rs13058191
All
−0.4
−0.3
−0.2
−0.1
0.0
MR leave−one−out sensitivity analysis for
'GCST90274762.csv' on 'Esophagus Cancer'
