# Supplementary material for: Exploring the role of adenosine deaminase in esophageal cancer and its potential for traditional Chinese medicine intervention
Source: Front Mol Biosci. 2026 Apr 23;13:1798177. doi: 10.3389/fmolb.2026.1798177 (PMC13149097; doi:10.3389/fmolb.2026.1798177)
Supplement: Supplementary file 1 [file DataSheet3.zip › Data Sheet 3.PDF]

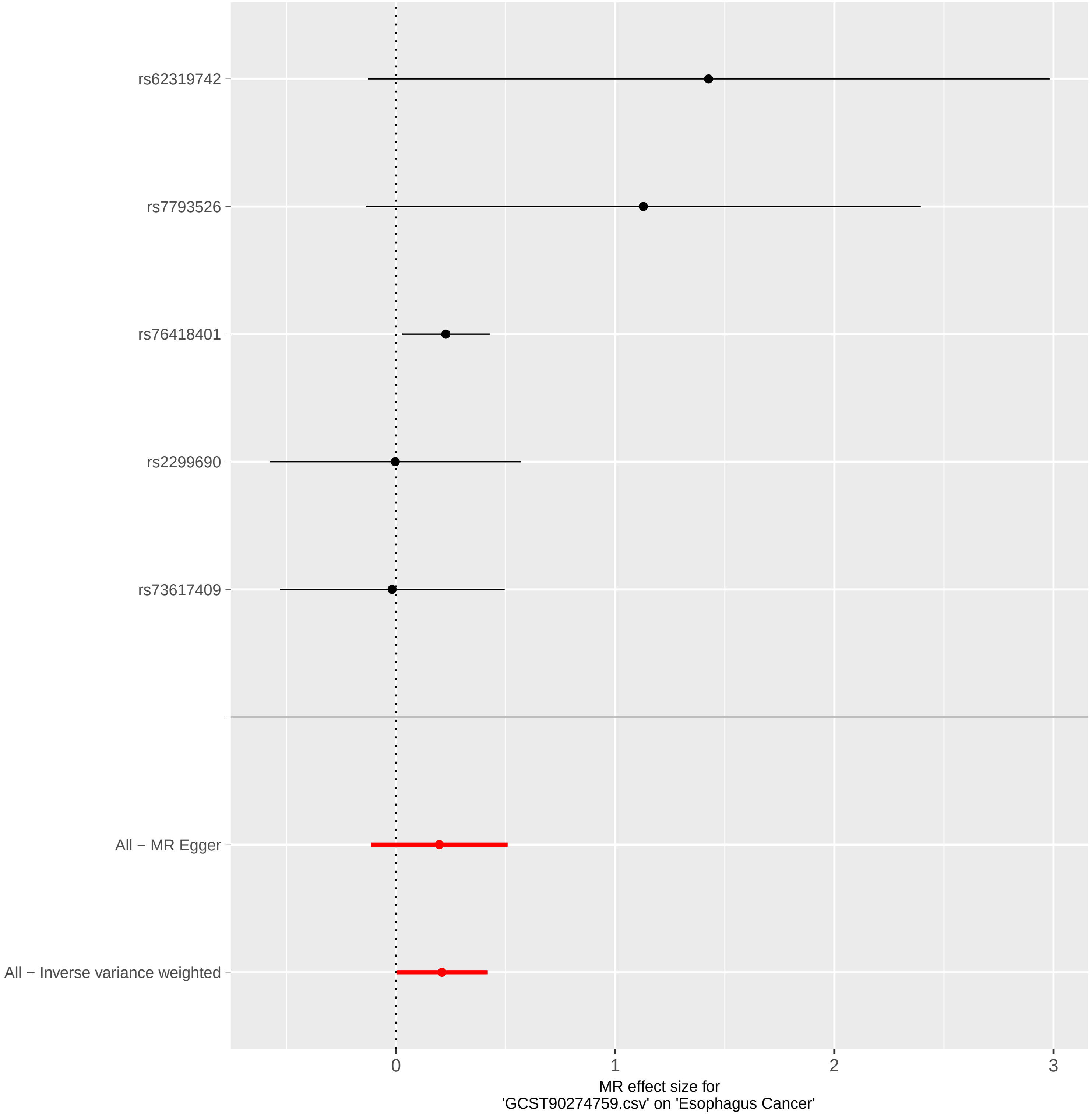

rs62319742
rs7793526
rs76418401
rs2299690
rs73617409
All − MR Egger
All − Inverse variance weighted
0
1
2
3
MR effect size for
'GCST90274759.csv' on 'Esophagus Cancer'

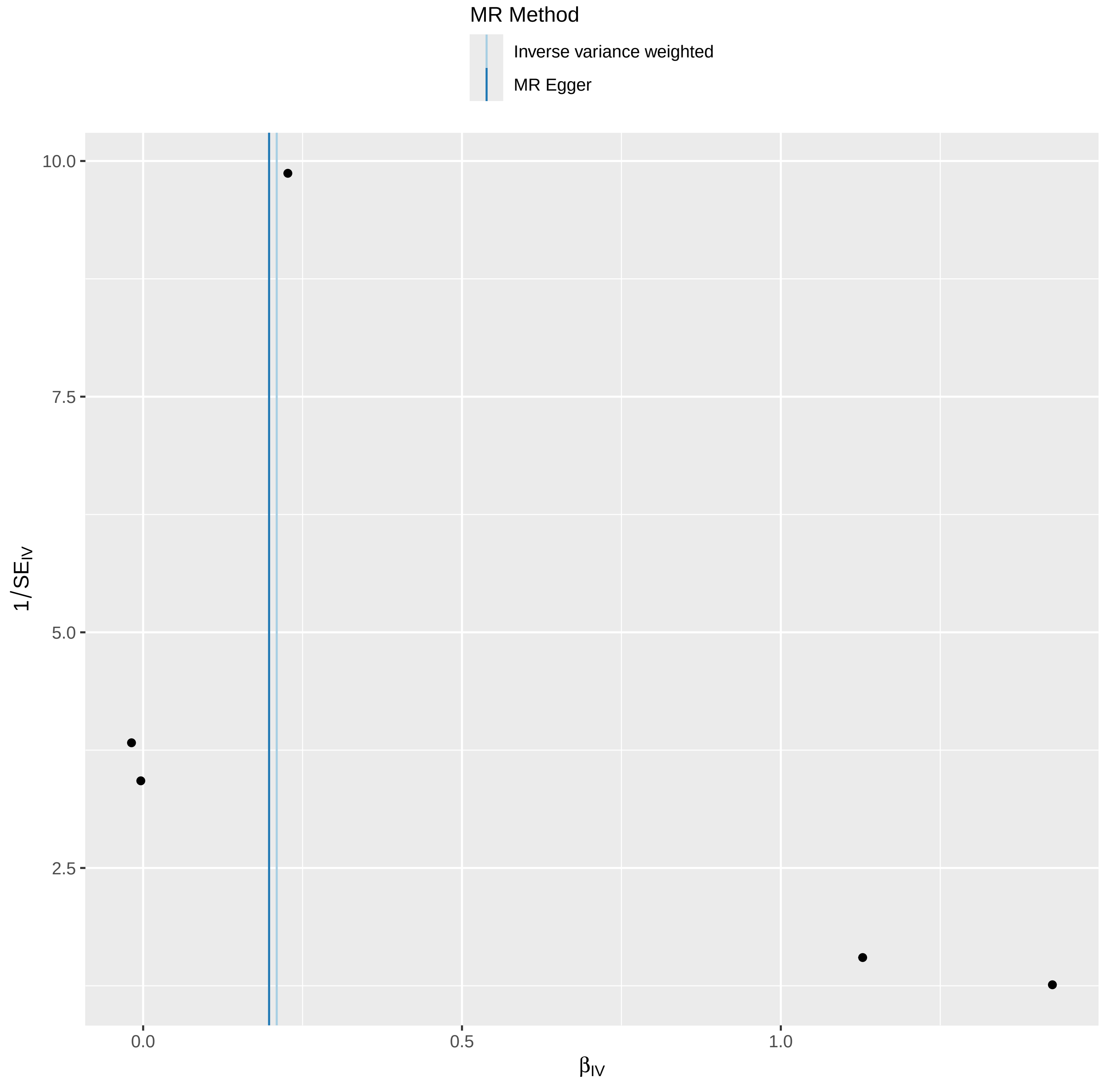
MR Method
Inverse variance weighted
MR Egger
10.0
7.5
5.0
2.5
0.0
0.5
1.0
1/SE_IV
β_IV

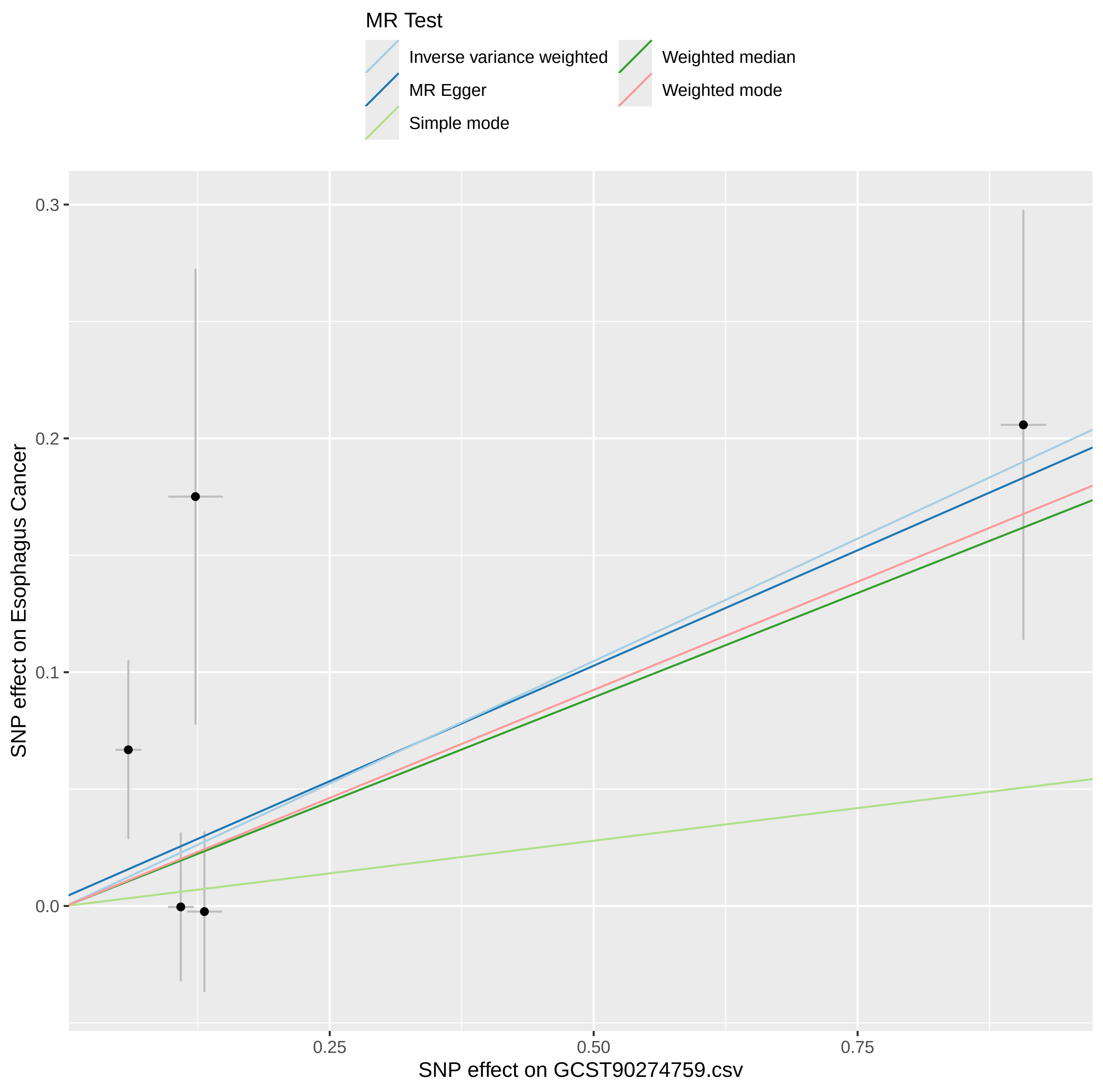

MR Test
Inverse variance weighted
MR Egger
Simple mode
Weighted median
Weighted mode
0.3
0.2
0.1
0.0
0.25
0.50
0.75
SNP effect on Esophagus Cancer
SNP effect on GCST90274759.csv

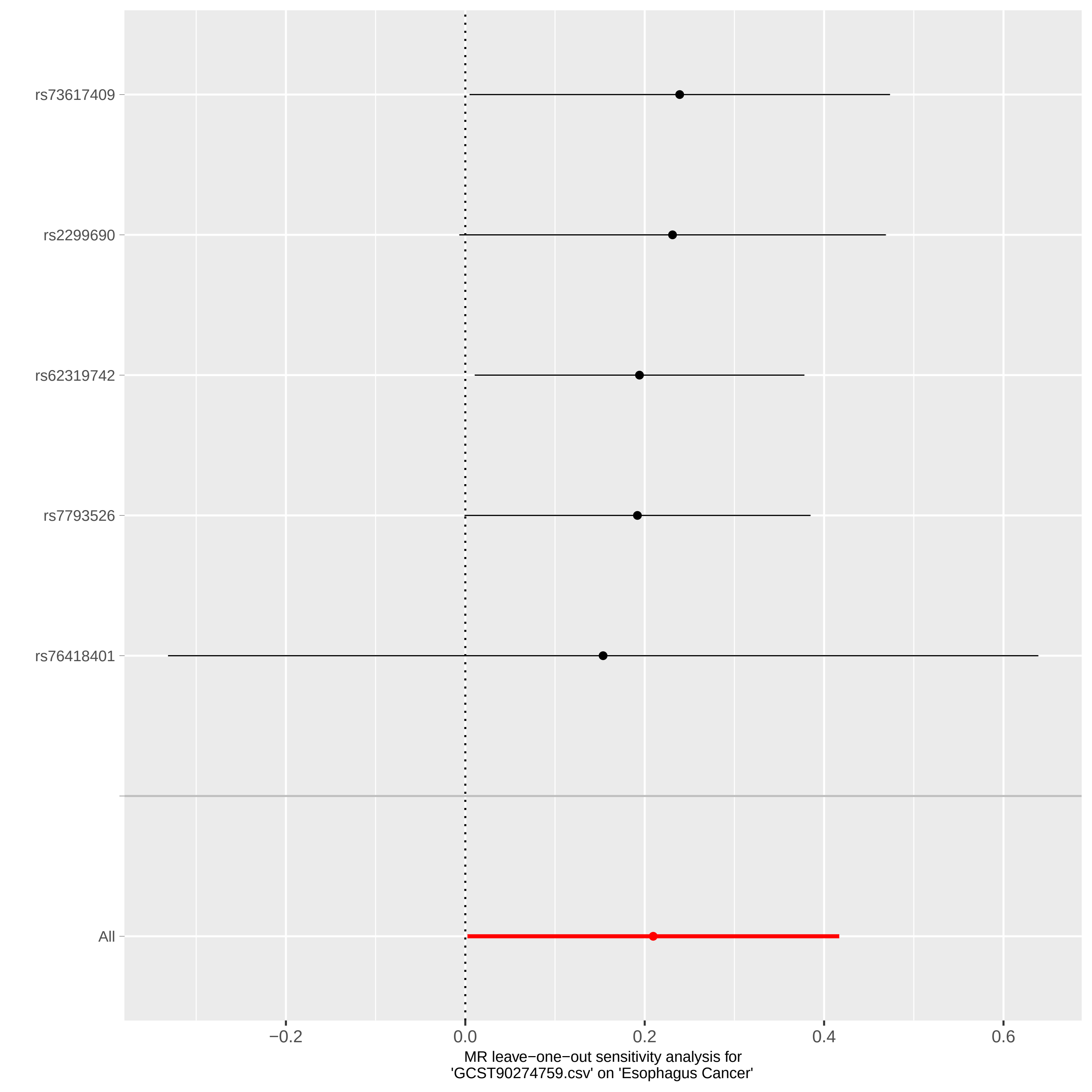

rs73617409
rs2299690
rs62319742
rs7793526
rs76418401
All
−0.2
0.0
0.2
0.4
0.6
MR leave−one−out sensitivity analysis for
'GCST90274759.csv' on 'Esophagus Cancer'
