# Supplementary material for: Exploring the role of adenosine deaminase in esophageal cancer and its potential for traditional Chinese medicine intervention
Source: Front Mol Biosci. 2026 Apr 23;13:1798177. doi: 10.3389/fmolb.2026.1798177 (PMC13149097; doi:10.3389/fmolb.2026.1798177)
Supplement: Supplementary file 1 [file DataSheet3.zip › Data Sheet 7.PDF]

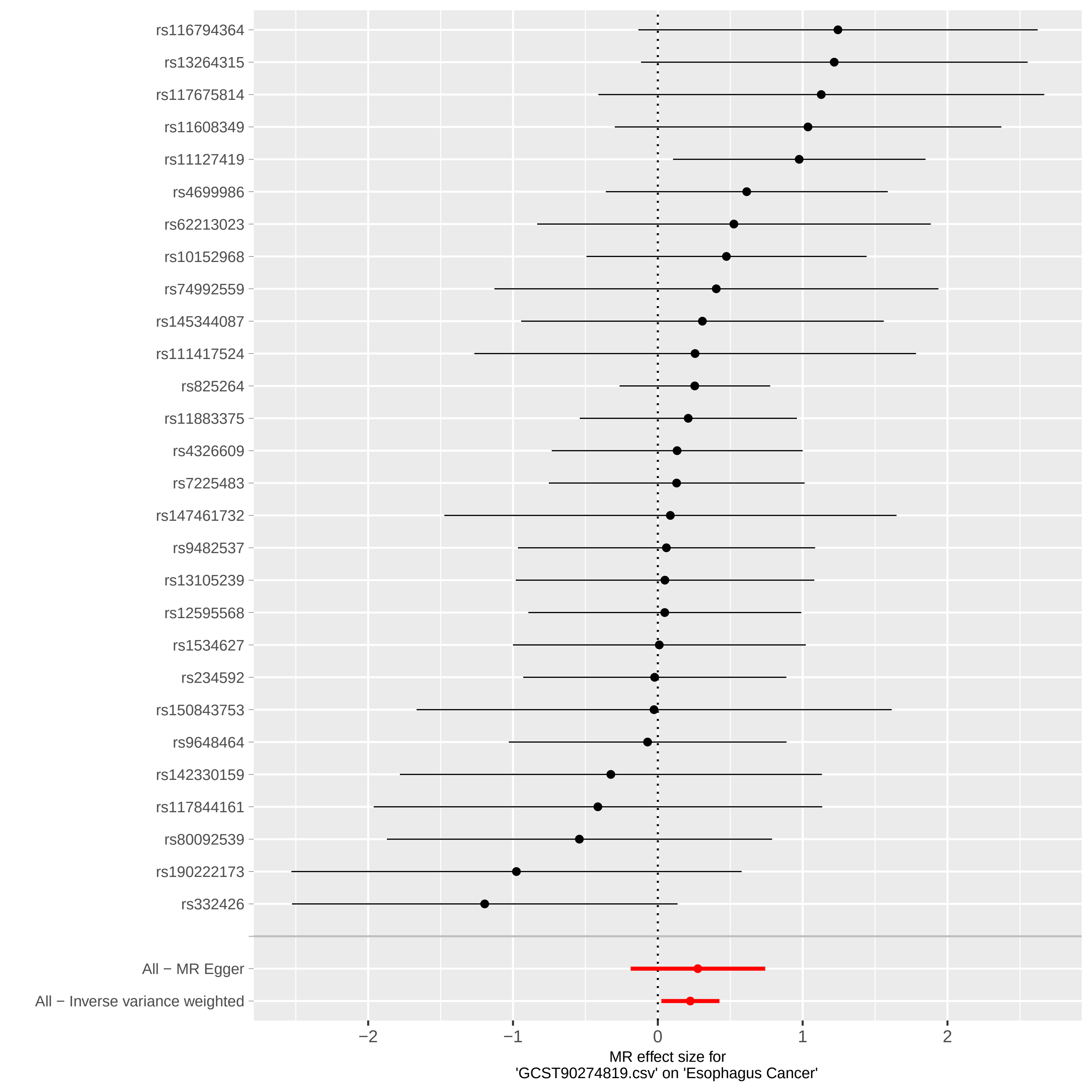
rs116794364
rs13264315
rs117675814
rs11608349
rs11127419
rs4699986
rs62213023
rs10152968
rs74992559
rs145344087
rs111417524
rs825264
rs11883375
rs4326609
rs7225483
rs147461732
rs9482537
rs13105239
rs12595568
rs1534627
rs234592
rs150843753
rs9648464
rs142330159
rs117844161
rs80092539
rs190222173
rs332426
All − MR Egger
All − Inverse variance weighted
−2
−1
0
1
2
MR effect size for
'GCST90274819.csv' on 'Esophagus Cancer'

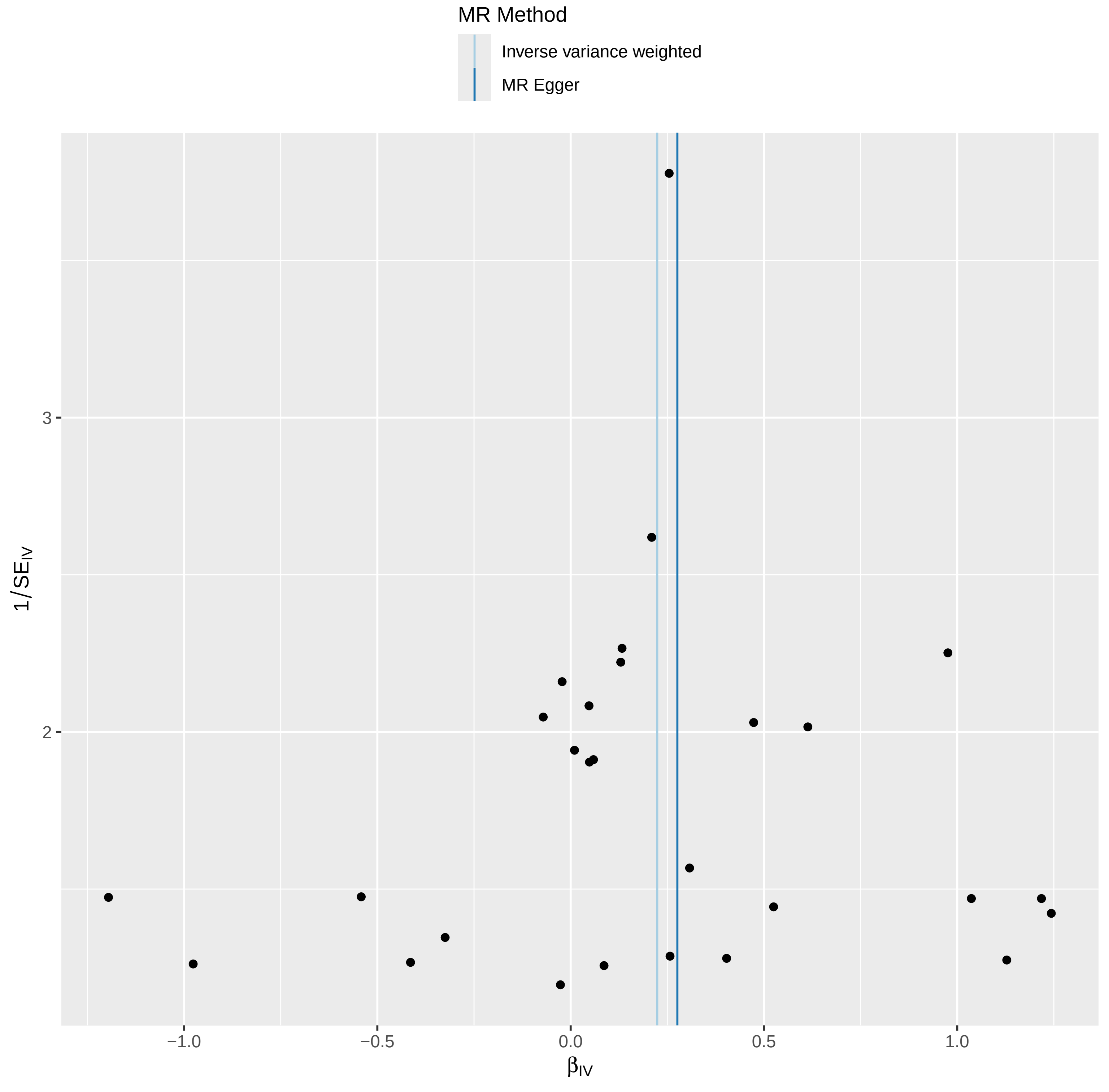
MR Method
Inverse variance weighted
MR Egger
1/SE_IV
3
2
β_IV
−1.0
−0.5
0.0
0.5
1.0

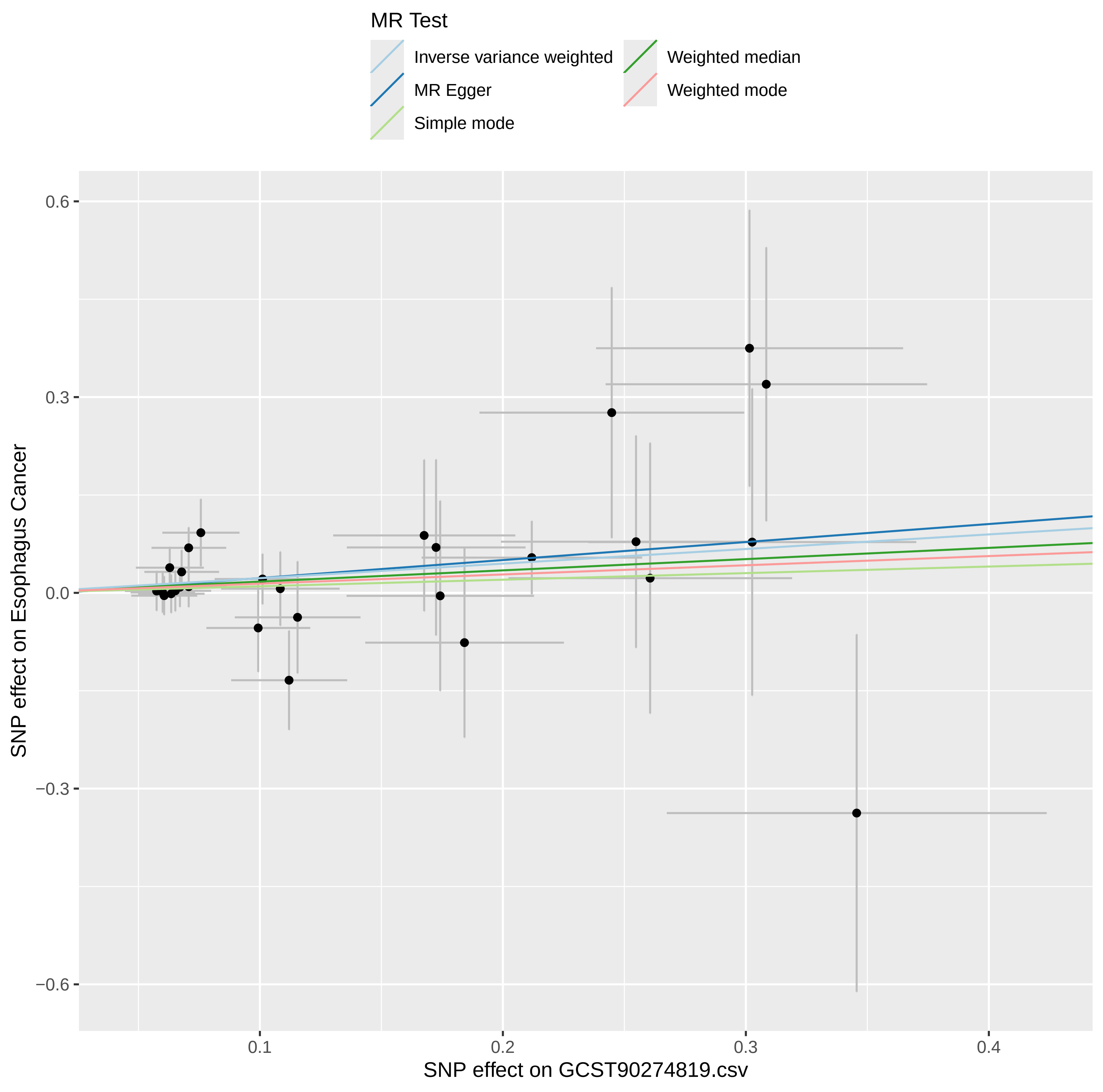

MR Test
Inverse variance weighted
MR Egger
Simple mode
Weighted median
Weighted mode
0.6
0.3
0.0
−0.3
−0.6
0.1
0.2
0.3
0.4
SNP effect on Esophagus Cancer
SNP effect on GCST90274819.csv

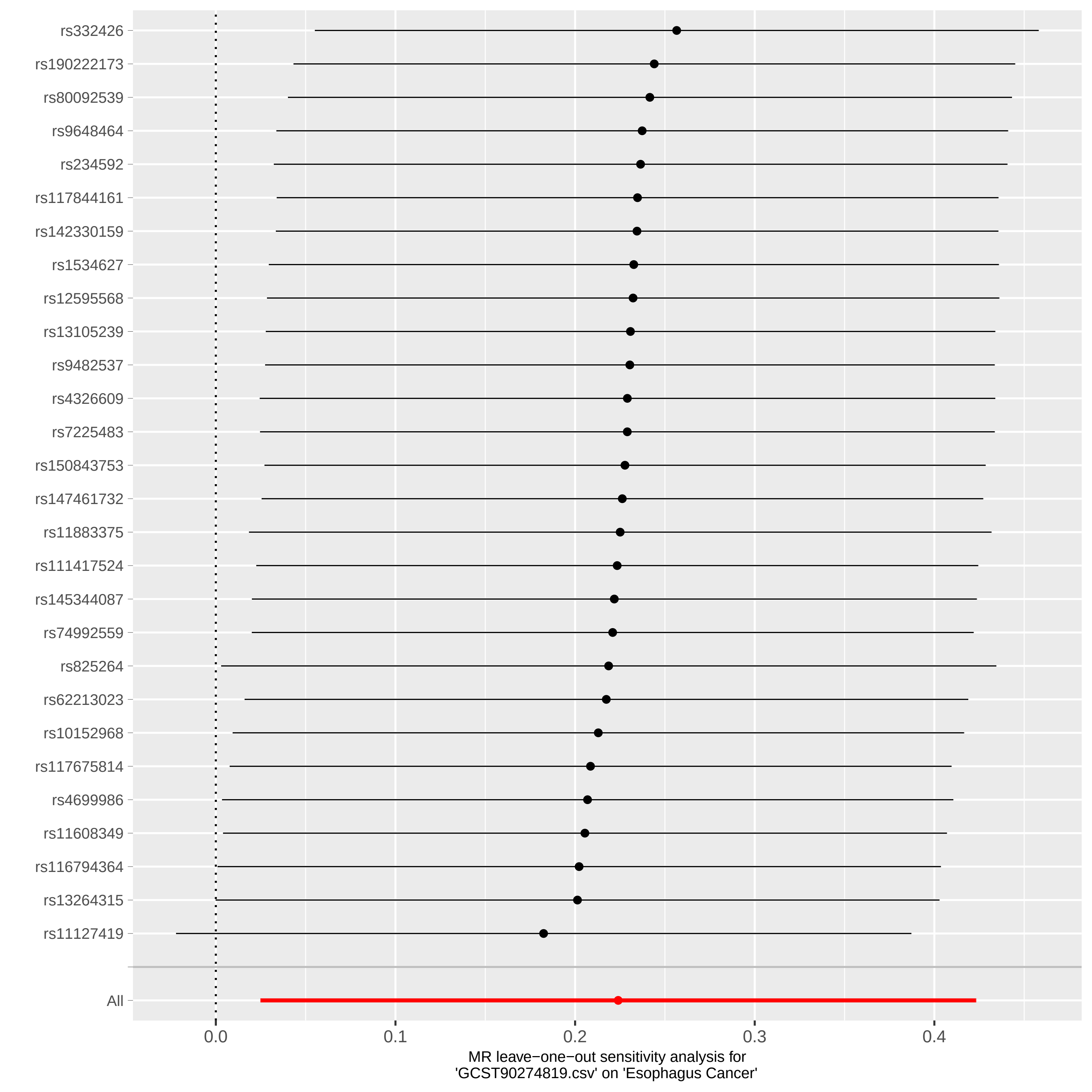
rs332426
rs190222173
rs80092539
rs9648464
rs234592
rs117844161
rs142330159
rs1534627
rs12595568
rs13105239
rs9482537
rs4326609
rs7225483
rs150843753
rs147461732
rs11883375
rs111417524
rs145344087
rs74992559
rs825264
rs62213023
rs10152968
rs117675814
rs4699986
rs11608349
rs116794364
rs13264315
rs11127419
All
0.0
0.1
0.2
0.3
0.4
MR leave−one−out sensitivity analysis for
'GCST90274819.csv' on 'Esophagus Cancer'
